# Saturated vs. unsaturated hydrocarbon interactions with carbon nanostructures

**DOI:** 10.3389/fchem.2014.00075

**Published:** 2014-09-03

**Authors:** Deivasigamani Umadevi, G. Narahari Sastry

**Affiliations:** Centre for Molecular Modeling, CSIR - Indian Institute of Chemical TechnologyHyderabad, India

**Keywords:** graphene, carbon nanotube, DFT, noncovalent interactions, saturated hydrocarbons, unsaturated hydrocarbons

## Abstract

The interactions of various acyclic and cyclic hydrocarbons in both saturated and unsaturated forms with the carbon nanostructures (CNSs) have been explored by using density functional theory (DFT) calculations. Model systems representing armchair and zigzag carbon nanotubes (CNTs) and graphene have been considered to investigate the effect of chirality and curvature of the CNSs toward these interactions. Results of this study reveal contrasting binding nature of the acyclic and cyclic hydrocarbons toward CNSs. While the saturated molecules show stronger binding affinity in acyclic hydrocarbons; the unsaturated molecules exhibit higher binding affinity in cyclic hydrocarbons. In addition, acyclic hydrocarbons exhibit stronger binding affinity toward the CNSs when compared to their corresponding cyclic counterparts. The computed results excellently corroborate the experimental observations. The interaction of hydrocarbons with graphene is more favorable when compared with CNTs. Bader's theory of atoms in molecules has been invoked to characterize the noncovalent interactions of saturated and unsaturated hydrocarbons. Our results are expected to provide useful insights toward the development of rational strategies for designing complexes with desired noncovalent interaction involving CNSs.

## Introduction

Carbon nanomaterials such as graphene and carbon nanotubes (CNTs) have been emerged as the promising materials for bio-medical applications due to their unique physical and chemical properties (Niyogi et al., 2002; Rao et al., 2009; De Volder et al., 2013). They have also been considered as potential candidates in designing of advanced functional materials for sensors, energy storage devices, fuel cells and electronics (Kumar et al., 2011). Owing to their exceptional properties and nanoscale dimensions, these nanomaterials provide great opportunities to mimic the single cells with the aim of designing chips that are as efficient as cells (Andersson and Van Den Berg, 2004). The intense sensitivity of carbon nanostructures (CNSs) toward doping and changes in the chemical environment make them suitable for sensors (Kong et al., 2000; Barone et al., 2005).

The noncovalent interactions of the CNSs have been widely recognized as they are essential to appreciate various applications of CNSs in biology and materials science (Chen et al., 2003; Zhao and Stoddart, 2009; Mahadevi and Sastry, 2013). The noncovalent interactions of CNSs such as cation−π, π − π, and CH···π with various metal ions, bio-molecules, and small molecules have been analyzed in several theoretical studies (Umadevi and Sastry, 2011a,b, 2012, 2013, 2014). Noncovalent interactions are ubiquitous and central in several areas of contemporary scientific interest. The influence of factors such as size, solvation and curvature of the systems on their noncovalent interactions has been studied extensively (Priyakumar et al., 2004; Rao et al., 2008; Vijay and Sastry, 2008; Premkumar et al., 2012). Recent reviews have highlighted the importance of noncovalent interactions in various fields (Wheeler, 2012; Mahadevi and Sastry, 2013). The π − π stacking interactions of CNSs with aromatic molecules are central in explaining the biological application of the CNSs. In addition to the π − π interactions which have been widely studied (Mahadevi et al., 2010; Chourasia et al., 2011), the XH···π interactions of the CNSs have also gained recent research interest. Kar et al. have studied the magnitude and nature of interactions between CNTs and aromatic systems (Kar et al., 2008). Schreiner and co-workers have shown from their studies that σ/σ and π/π interactions are equally important and thus insisted the possibility of the existence of multilayered graphanes (Fokin et al., 2011). The importance of noncovalent interactions in understanding various applications of CNSs has been elaborately discussed in a recent accounts (Umadevi et al., 2014).

The physical and chemical properties of the CNSs, critically depend on their dimensionality. Graphene consists of a planar arrangement of carbon atoms packed in a two-dimensional hexagonal lattice. The extended π-network in the 2D graphene is the basic building block for CNSs of various dimensions, for instance 3D graphite, which is formed from the stacked layers of graphene, 1D CNTs which is basically the rolled graphene sheet and the 0D fullerene is formed by wrapping the graphene sheet. We have shown by a series of studies that the planar graphene shows stronger binding affinity than the curved CNTs toward noncovalent interactions (Umadevi and Sastry, 2011b, 2013, 2014). Besides, comparing and contrasting the binding affinity of CNTs of different chirality and curvature is interesting in its own right. Subramanian and co-workers have made seminal contributions to understand the effect of curvature and chirality of CNSs on binding with various bio-molecules (Balamurugan et al., 2011; Ravinder et al., 2012; Balamurugan and Subramanian, 2013). It has also been shown from our group that the zigzag CNTs show stronger propensity to bind with various aromatic molecules and bio-molecules than the armchair CNTs (Umadevi and Sastry, 2013).

Even though CNSs have shown to be potential for a wide range of applications, their chemical inertness and insoluble nature are the major restrictions for their thriving applications. Covalent and noncovalent functionalization of CNSs serves as a useful tool to overcome these limitations and provide much easier manipulation of the CNSs. Kim and co-workers have comprehensively reviewed the covalent and noncovalent functionalization of graphene in a recent literature (Georgakilas et al., 2012). Noncovalent functionalization emerged as the most efficient way of functionalizing the CNSs without disturbing their honeycomb carbon network. Li et al. have shown the possibility of using noncovalent functionalization of alkali metals to separate semiconducting CNTs from metallic CNTs (Li et al., 2013). A recent study describes the strength and importance of the noncovalently functionalized graphene by polymeric adsorbates (Mann and Dichtel, 2013). Thus, the noncovalent interaction of CNSs is one of the very actively examined concepts in modern nanochemistry as it is believed to open the path toward various innovative applications.

The adsorption and separation of various gas mixtures play significant role in various disciplines. CNSs have also been used in the adsorption and separation of olefin/paraffin mixtures which is one of the key processes in the chemical and petrochemical industries (Cruz and Mota, 2009; Albesa et al., 2011). Jiang et al. studied the adsorption and separation of linear and branched alkanes on CNT bundles (Jiang et al., 2005). Clearly, there is a need to better understand the difference in the binding strength of these saturated and unsaturated hydrocarbons (Premkumar et al., 2014). Besides, the study of alkane and alkene interactions with CNSs has been invoked to throw light on the π − π and CH···π interactions which is of fundamental importance in the contemporary nanoscience literature. The current study aims to endow with a comprehensive and comparative analysis of the interactions of various saturated and unsaturated hydrocarbons with CNSs. The different possible orientations and their binding strength have been addressed. We have also studied the effect of curvature and chirality of the carbon materials on the binding strength. Comparisons have been given with experimental data whenever these are available. The nature of line critical points (LCPs) between the hydrocarbons and CNSs in the saturated and unsaturated hydrocarbons has been characterized with the help of the Bader's theory of atoms in molecules (Bader, 1990; Cortés-Guzmán and Bader, 2005).

## Computational methods

**Figure 1:**
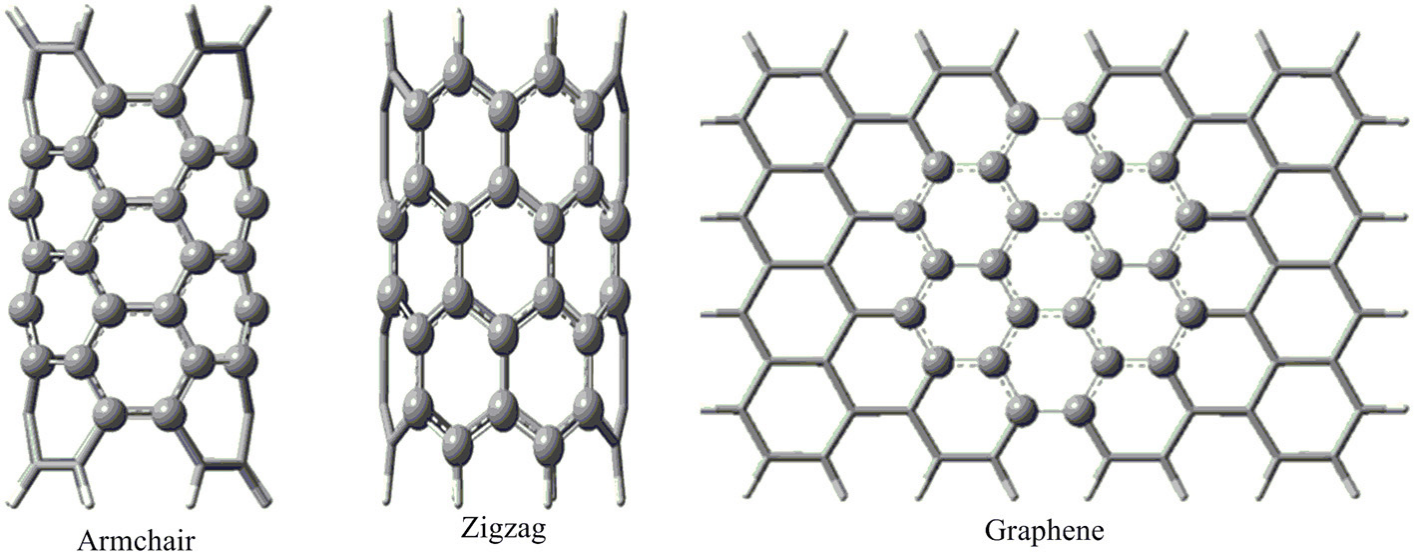
**The atoms shown by the ball-and-stick model were considered for the higher layer and the rest of the atoms shown by the tube model were considered for the lower layer in the ONIOM calculation**.

Geometry optimization of all the model structures considered have been done by employing a two-layer ONIOM approach as implemented in Gaussian 09 (Frisch et al., 2009). The hydrocarbon molecules considered and a seven ring fragment in the CNSs, resembling coronene (24 atoms) has been considered as the high layer and the rest of the system has been considered as the low layer as shown in the Figure 1. The dangling bonds at the truncated ends of the CNSs were passivated with hydrogen atoms to avert spurious end effects.

The stationary points obtained have been characterized as minima by verifying the presence of all real frequencies. All the ONIOM geometry optimizations have been done at (M06–2X/6–31G^*^: AM1) level of theory. While many popular functionals of the density functional theory (DFT) be inadequate to model the noncovalent interactions, the de novo parameterized M06-2X functionals of Zhao and Truhlar has been proven to be suitable (Zhao and Truhlar, 2008).

The ONIOM (QM:QM) models have been effectively employed to study various noncovalent interactions of CNSs in the earlier studies (Umadevi and Sastry, 2011b, 2012, 2013, 2014). However, in order to ensure the reliability of ONIOM approach for the accurate description of energies, full geometry optimization of CNT(4,4) has been done at M06-2X/6-31G^*^ level and the results obtained were compared with the ONIOM results. It is found from Table 1 that the results obtained in the ONIOM approach are in good concurrence with the full optimization. Binding energy (BE) has been calculated by means of the super molecule approach, where BE is the difference between sum of the total energies of the parent CNS (E_CNS_) and the hydrocarbon (E_X_) and the total energy of the complex (E_CNS_X_) as shown in equation 1.

(1) $$\text{BE}=({\text{E}}_{\text{CNS}}+{\text{E}}_{\text{X}})-{\text{E}}_{\text{CNS}\_\text{X}}$$

**Table 1:** **BE (kcal/mol) of the hydrocarbons with CNT (4,4) obtained using ONIOM approach and full optimization**.

**Molecules**	M06-2X/6-31G^*^//ONIOM (M06-2X/6-31G^*^: AM1)	M06-2X/6-31G^*^
	**BE**	**BE**
A2	−3.76	−3.74
A4	−6.19	−6.15
A6	−9.16	−9.24
CA4	−6.51	−6.56
CA6	−4.72	−4.72
CA10	−8.03	−7.99
E2	−3.30	−3.29
E4	−5.84	−5.89
E6	−7.96	−7.98
CE4	−6.10	−6.09
CE6	−6.21	−6.24
CE10	−9.19	−9.11

The energy values thus obtained have been fine tuned by single point calculations at M06-2X/6-311G^**^ level. Electron density at the LCPs in all the structures has been mapped using the AIM2000 program (Biegler-König, 2000, 2001; Biegler-König and Schönbohm, 2002).

### Model systems considered in the study

**Figure 2:**
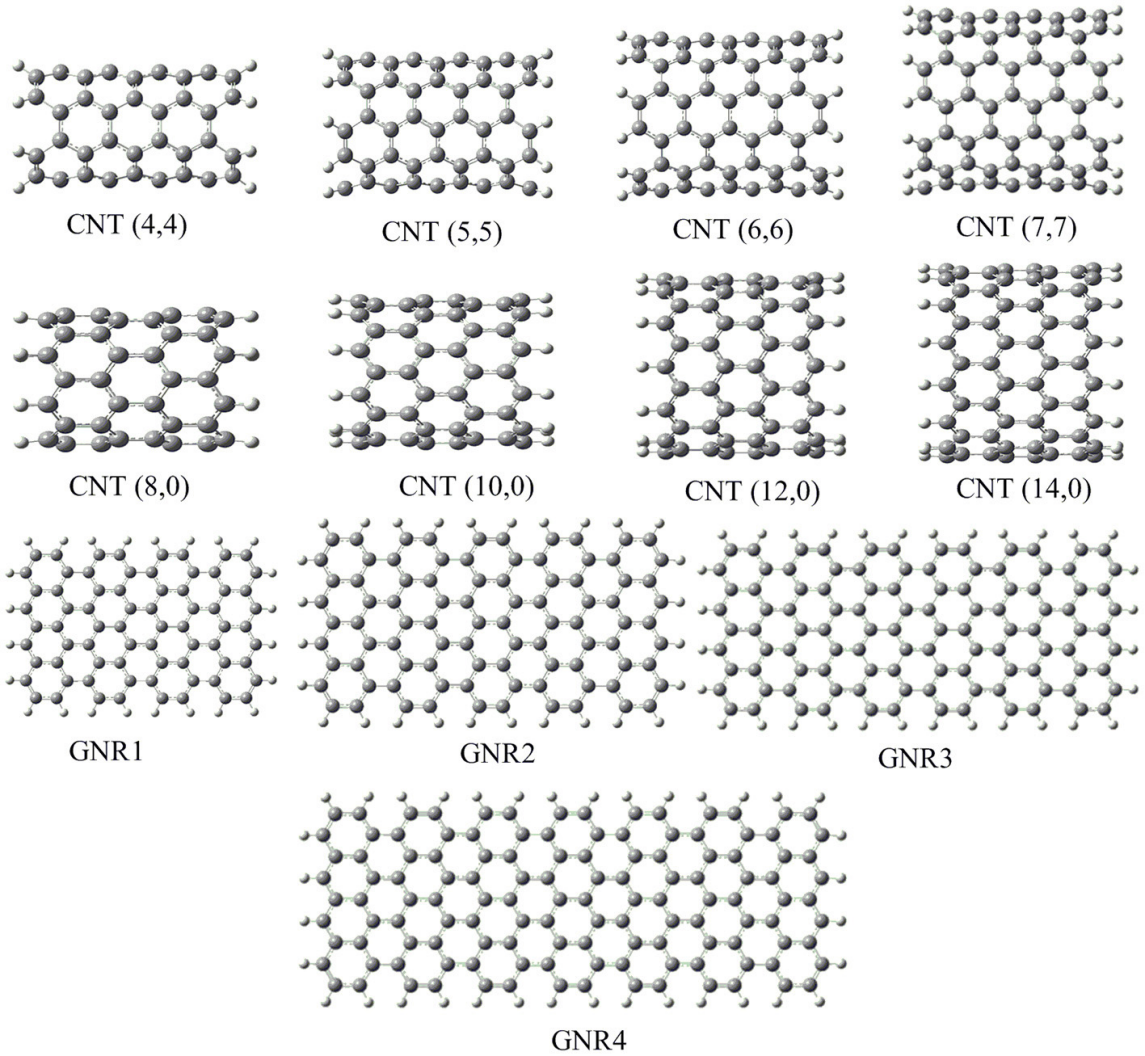
**Model systems used to represent the carbon nanostructures**.

**Figure 3:**
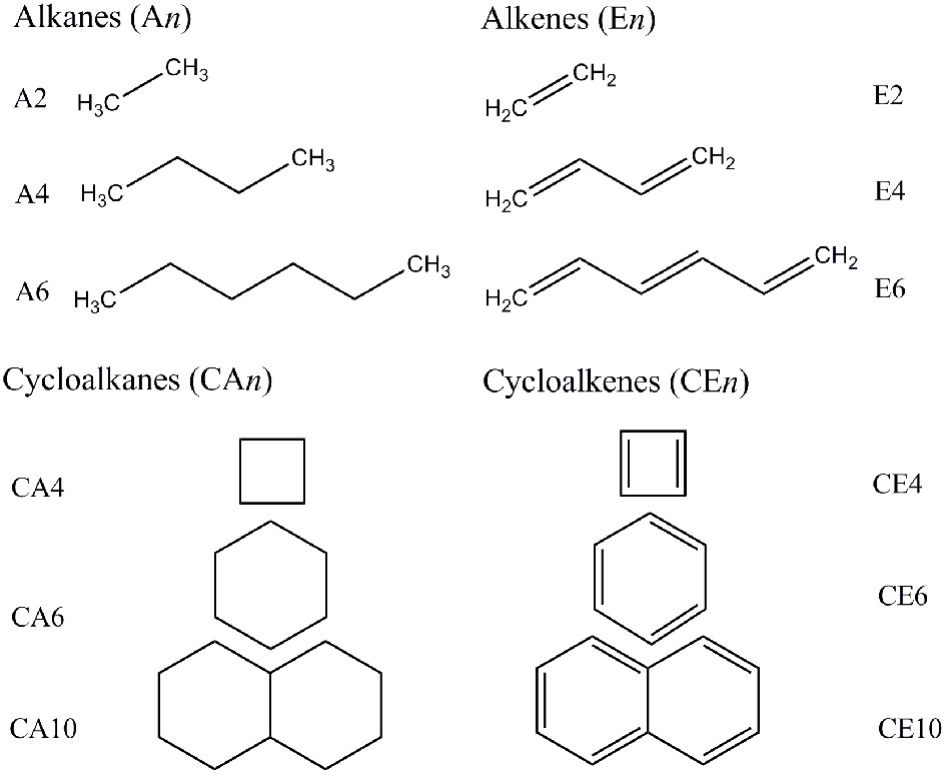
**Acylic and acylic hydrocarbons considered in this study**.

We adopted cluster models for the first-principles calculations to represent the structure of CNSs such as graphene and CNTs as shown in Figure 2. Armchair CNTs with various diameters such as CNT(4,4), CNT(5,5), CNT(6,6), CNT(7,7), and zigzag CNTs such as CNT(8,0), CNT(10,0), CNT(12,0), CNT(14,0) have been considered. Besides, graphene nanoribbons (GNRs) such as GNR1, GNR2, GNR3, and GNR4 have been modeled. Thus, the eclectic model systems considered will help to determine the effect of chirality and curvature of the CNSs toward binding with the hydrocarbons. The model systems used to represent the hydrocarbons have been given in Figure 3. A set of acyclic hydrocarbons in both saturated and unsaturated forms and a set of cyclic hydrocarbons in both saturated and unsaturated forms have been considered to study their interaction with CNSs. The nomenclature used in this discussion is as follows; acyclic saturated hydrocarbons (A*n*), acyclic unsaturated hydrocarbons (E*n*), cyclic saturated hydrocarbons (CA*n*) and cyclic unsaturated hydrocarbons (CE*n*), where *n* denotes the number of carbon atoms.

## Results and discussion

The discussion is focused on the comparison of the binding affinity of the saturated and unsaturated hydrocarbons. The binding affinity of the cyclic hydrocarbons has also been compared with the corresponding acyclic hydrocarbons. This is followed by the portrayal of the effect of chirality and curvature of the CNSs toward the hydrocarbon binding. We also discuss the results of AIM analysis which provides further insight to the binding of saturated and unsaturated hydrocarbons. A qualitative comparison has been made with our results and the available experimental results.

### Saturated vs. unsaturated hydrocarbons

**Figure 4:**
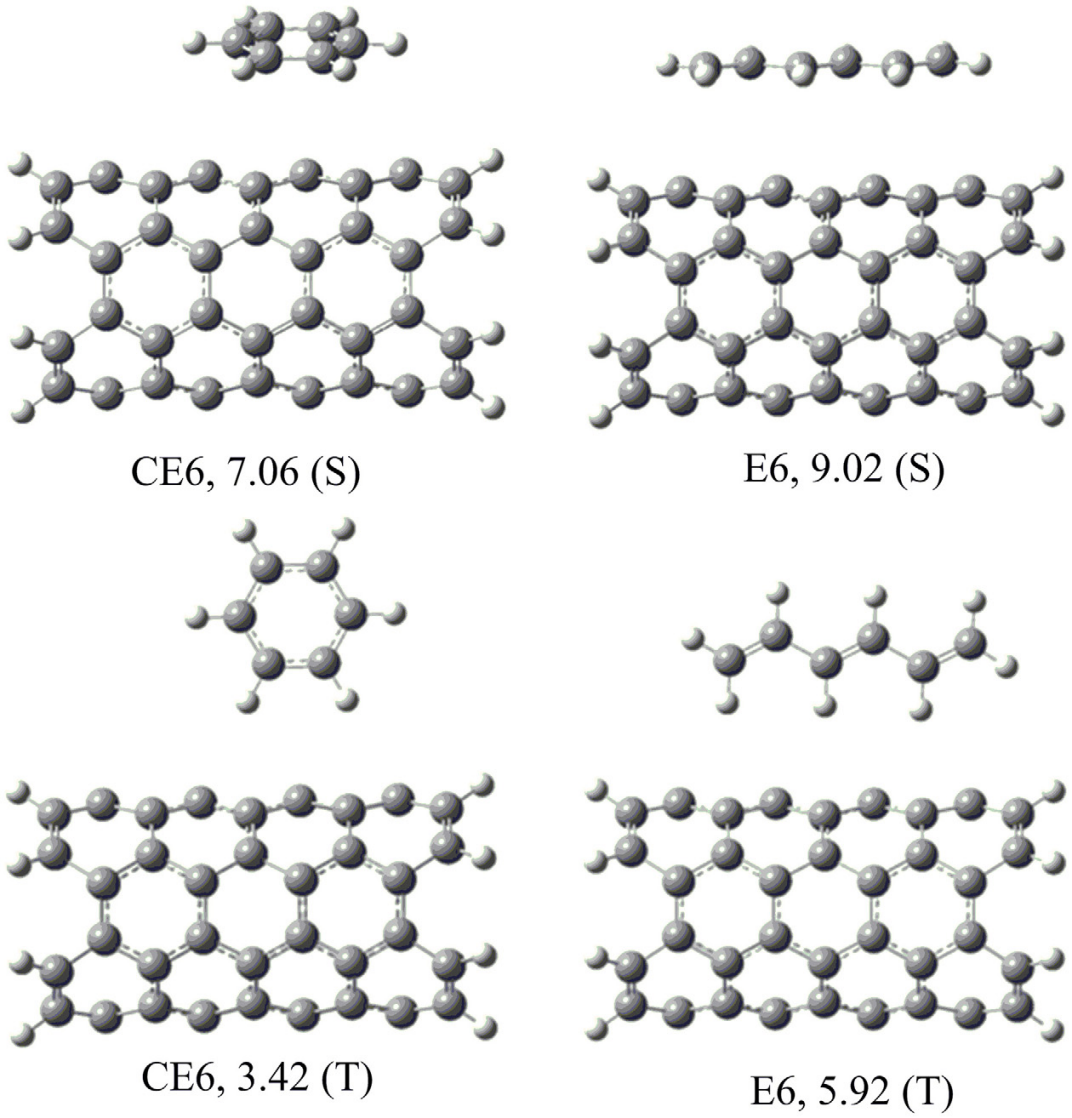
**Possible orientations (S and T) of the unsaturated hydrocarbons on CNSs and their binding energies (kcal/mol)**.

A systematic analysis has been done to understand the interaction of hydrocarbons with the CNSs. Before proceeding with all CNSs, a preliminary study has been done, where various possible orientations of all the hydrocarbons considered with CNT(4,4). The most stable orientations observed from the preliminary study have been considered for further study in all other CNSs. For instance, E*n* and CE*n* hydrocarbons orients themselves in stacked (*S*) or T-shaped (*T*) orientations with respect to the CNSs, which makes them interact by CH···π or π − π mode of interactions, respectively (Figure 4). Our results show that *S* orientations are more stable than the *T* orientations, in both the cases of E*n* and CE*n*. A similar analysis has been done for all the other hydrocarbons with the CNT(4,4) and the results have been given in the Supplementary Material. The best possible orientations obtained from the above analysis have been considered for further study.

#### Acyclic hydrocarbons—saturated vs. unsaturated

The BE of acyclic hydrocarbons such as ethane (A2), n-butane (A4), and n-hexane (A6) have been calculated with armchair CNTs, zigzag CNTs and GNRs and the values are listed in Table 2. It is evident from the table that, as the size of the hydrocarbon increases the BE is also increasing. The increase in the BE energy is more prominent in the case of graphene than the CNTs. In order to compare their unsaturated counterparts, we have calculated the BE of ethylene (E2), 1,3-butadiene (E4), and 1,3,5-hexatriene (E6) with all the considered CNSs. As observed in the case of A*n*, the BE increases as the size of the E*n* increases and the increase is more dramatic in the case of graphene. An interesting observation is that, the binding affinity of A*n* has been found to be higher than the corresponding E*n*. The same trend has been noted for both CNTs and GNRs; however, the difference obtained in their BE is much lower in the case of graphene. In general, alkane molecules interact with the CNTs via CH···π interaction where as alkenes interact by π − π interaction. Our results indicate that in the case of acyclic hydrocarbons, the saturated A*n* molecules bind more strongly than the unsaturated hydrocarbons E*n*.

**Table 2:** BE (kcal/mol) of CNSs with various acyclic saturated (A*n*) and unsaturated (E*n*) hydrocarbons at M06-2X/6-311G^**^//ONIOM (M06-2X/6-31G^*^: AM1) level.

**Armchair**			**Zigzag**			**Graphene**		
	**A*n***	**E*n***		**A*n***	**E*n***		**A*n***	**E*n***
**CNT(4,4)**			**CNT(8,0)**			**GNR1**		
*n* = 2	4.13	3.74	*n* = 2	4.91	3.70	*n* = 2	5.01	5.08
*n* = 4	6.82	6.59	*n* = 4	7.12	6.15	*n* = 4	9.48	8.97
*n* = 6	9.98	9.06	*n* = 6	10.05	8.70	*n* = 6	13.55	13.10
**CNT (5,5)**			**CNT(10,0)**			**GNR2**		
*n* = 2	4.44	3.85	*n* = 2	3.97	3.76	*n* = 2	5.01	5.01
*n* = 4	7.76	7.30	*n* = 4	7.42	6.51	*n* = 4	9.44	9.20
*n* = 6	10.40	9.39	*n* = 6	10.09	10.03	*n* = 6	13.48	12.60
**CNT (6,6)**			**CNT(12,0)**			**GNR3**		
*n* = 2	4.61	4.04	*n* = 2	4.22	3.86	*n* = 2	5.02	4.53
*n* = 4	7.00	5.74	*n* = 4	7.90	6.77	*n* = 4	9.45	8.98
*n* = 6	11.48	10.00	*n* = 6	11.00	10.67	*n* = 6	13.48	13.08
**CNT (7,7)**			**CNT(14,0)**			**GNR4**		
*n* = 2	4.34	4.09	*n* = 2	4.52	3.93	*n* = 2	5.00	5.14
*n* = 4	7.95	7.44	*n* = 4	8.26	7.79	*n* = 4	9.31	8.47
*n* = 6	11.52	10.20	*n* = 6	11.23	10.03	*n* = 6	13.42	12.63

**Figure 5:**
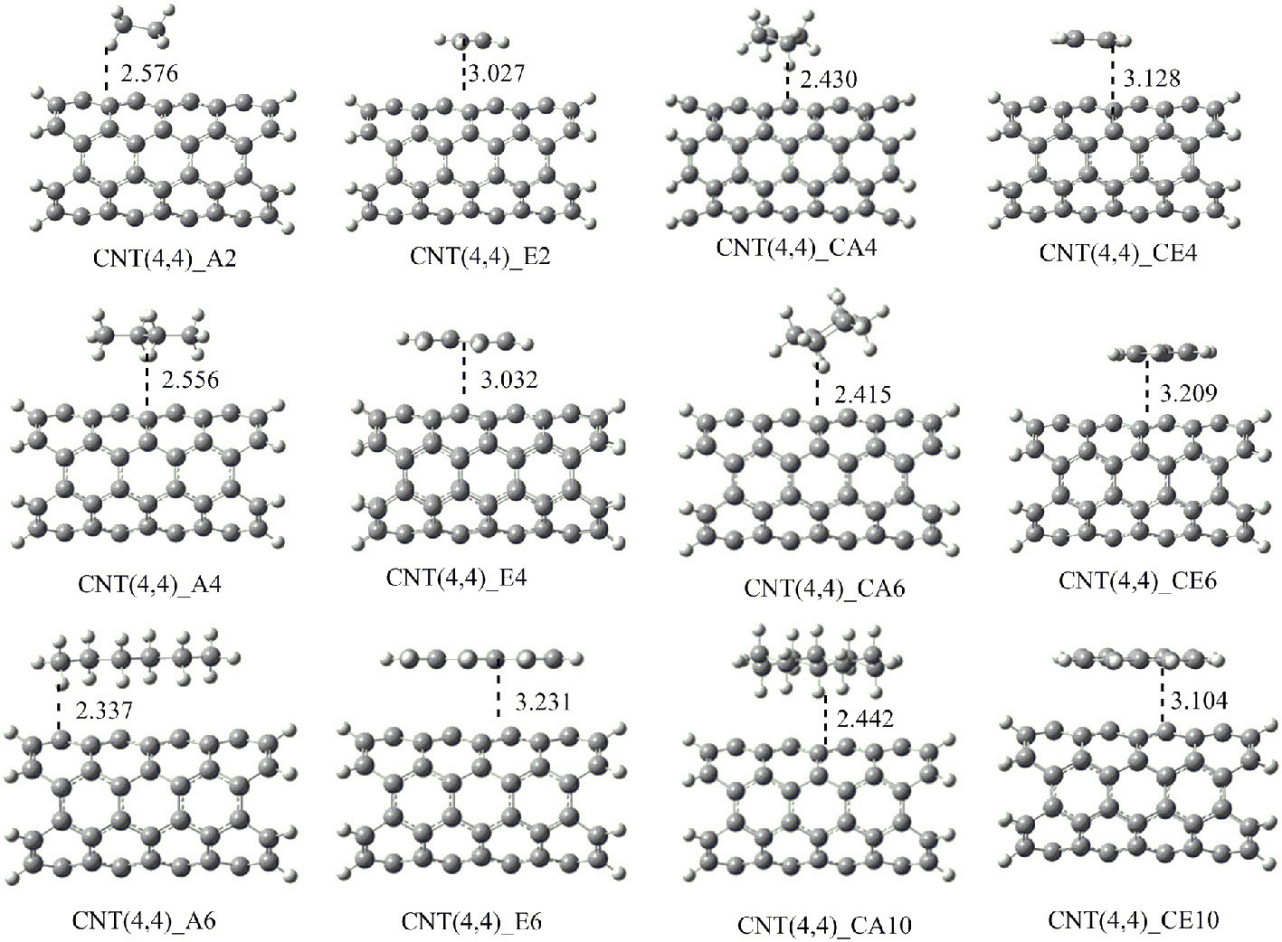
**Optimized geometries of the complexes CNT (4,4) with all the considered hydrocarbons**. The nearest distance between the monomers is given in Å.

The shortest distance between CNSs and the hydrocarbons (r) obtained in the geometry optimization of the CNS-A*n* and CNS-E*n* complexes have been listed in Table S1 (Supplementary Material). In the case of CNS-A*n* complexes, the distance is measured from the nearest H atom of the A*n* to the surface of the CNSs. For E*n* complexes, the distance is measured from the nearest C atom of the E*n* to the surface of the CNSs. It is observed from Table S1 that the A*n* hydrocarbons are at a distance of around 2.5Å from the CNS surface. And E*n* orient themselves from the CNSs surface at a distance of around 3.0Å. The representative optimized structures and the geometry parameters of the CNT-hydrocarbon complexes have been given in Figure 5 and the rest of the structures have been given in the Supplementary Material.

#### Cyclic hydrocarbons—saturated vs. unsaturated

We further focused on the BE of cyclic saturated hydrocarbons such as cyclobutane (CA4), cyclohexane (CA6), and decalin (CA10) with CNSs. The results obtained have been compared with that of the BE of their unsaturated counterparts such as cyclobutadiene (CE4), benzene (CE6), and naphthalene (CE10). Table 3 summarizes the BE values of both saturated and unsaturated cyclic hydrocarbons. It is obvious from the table that the BE increases as the size of the hydrocarbon increases in most of the complexes except in a few complexes of CNTs, where the BE of CE4 has been found to be more than the CE6 complexes. In contrast to the observation in acyclic hydrocarbons, the binding affinity of unsaturated CE*n* is more than that of its saturated counterpart CA*n*.

**Table 3:** BE (kcal/mol) of CNSs with various cyclic saturated (CA*n*) and unsaturated (CE*n*) hydrocarbons at M06-2X/6-311G^**^//ONIOM (M06-2X/6-31G^*^: AM1) level.

**Armchair**			**Zigzag**			**Graphene**		
	**CA*n***	**CE*n***		**CA*n***	**CE*n***		**CA*n***	**CE*n***
**CNT(4,4)**			**CNT(8,0)**			**GNR1**		
*n* = 4	5.92	6.79	*n* = 4	7.39	7.58	*n* = 4	7.19	9.01
*n* = 6	5.25	7.08	*n* = 6	5.84	7.80	*n* = 6	7.89	10.91
*n* = 10	9.18	10.69	*n* = 10	9.68	11.23	*n* = 10	13.55	17.44
**CNT (5,5)**			**CNT(10,0)**			**GNR2**		
*n* = 4	5.92	7.43	*n* = 4	6.89	7.18	*n* = 4	7.20	8.73
*n* = 6	6.60	7.32	*n* = 6	5.78	8.56	*n* = 6	8.01	10.44
*n* = 10	10.06	11.75	*n* = 10	10.78	12.59	*n* = 10	13.16	16.78
**CNT (6,6)**			**CNT(12,0)**			**GNR3**		
*n* = 4	6.47	6.98	*n* = 4	6.07	7.65	*n* = 4	7.20	9.00
*n* = 6	6.52	8.21	*n* = 6	6.96	8.72	*n* = 6	7.91	10.99
*n* = 10	9.28	12.10	*n* = 10	10.46	12.78	*n* = 10	13.48	17.43
**CNT (7,7)**			**CNT(14,0)**			**GNR4**		
*n* = 4	6.62	7.42	*n* = 4	6.83	8.06	*n* = 4	7.22	8.71
*n* = 6	6.31	8.06	*n* = 6	6.23	8.69	*n* = 6	7.96	11.04
*n* = 10	10.17	12.95	*n* = 10	10.65	13.53	*n* = 10	13.26	16.78

### Cyclic vs. acyclic hydrocarbons

**Figure 6:**
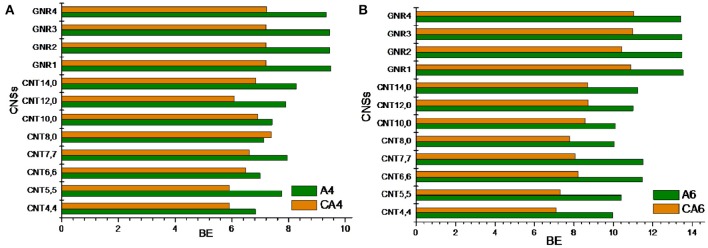
**Binding energy of cyclic vs. acyclic saturated hydrocarbons**. **(A)** n-butane vs. cyclobutane and **(B)** n-hexane vs. cyclohexane.

**Figure 7:**
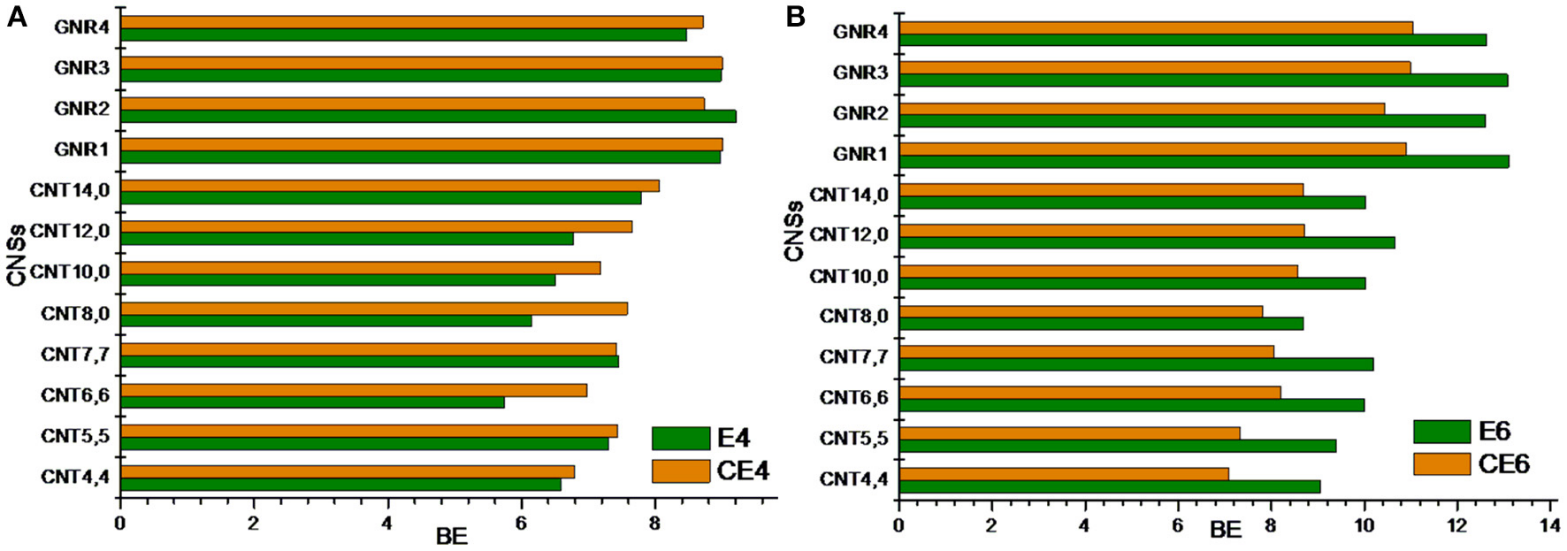
**Binding energy of cyclic vs. acyclic unsaturated hydrocarbons**. **(A)** 1,3 butadiene vs. cyclobutadiene and **(B)** 1,3,5 hexatriene vs. benzene.

In this section, we have compared the binding affinity of acyclic hydrocarbon with their cyclic counterparts toward the CNSs. For instance, we have considered A4 and A6 hydrocarbons in the acyclic set, which can be compared with that of the cyclic hydrocarbon CA4 and CA6. Similarly, in the unsaturated set E4 and E6 can have the cyclic counterparts as CE4 and CE6. Comparing and contrasting the binding affinity of these hydrocarbons will be interesting in its own right. Figures 6, 7 illustrate the binding affinity of the cyclic vs. acyclic hydrocarbons in their saturated and unsaturated forms, respectively. The cyclic and acyclic forms of the hydrocarbons with same number of carbon atoms have been compared. It is clear from the figures that the acyclic hydrocarbons show stronger binding affinity toward the CNSs than their cyclic counterparts. However, there is not much difference observed between the binding affinities of the E4 and CE4 complexes.

### Effect of chirality and curvature

**Figure 8:**
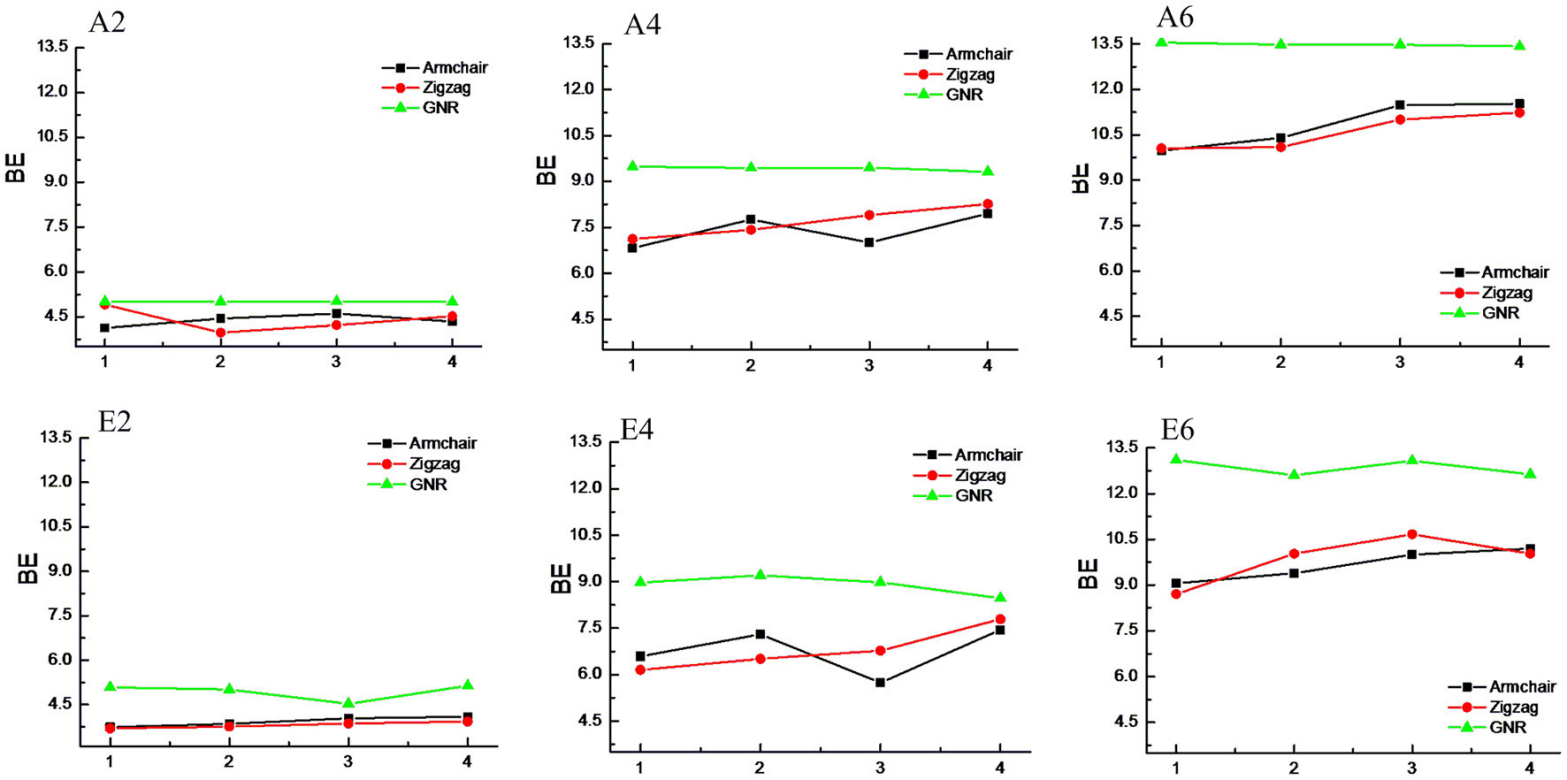
**Effect of curvature and chirality on the binding affinity of acyclic hydrocarbons with CNSs**.

**Figure 9:**
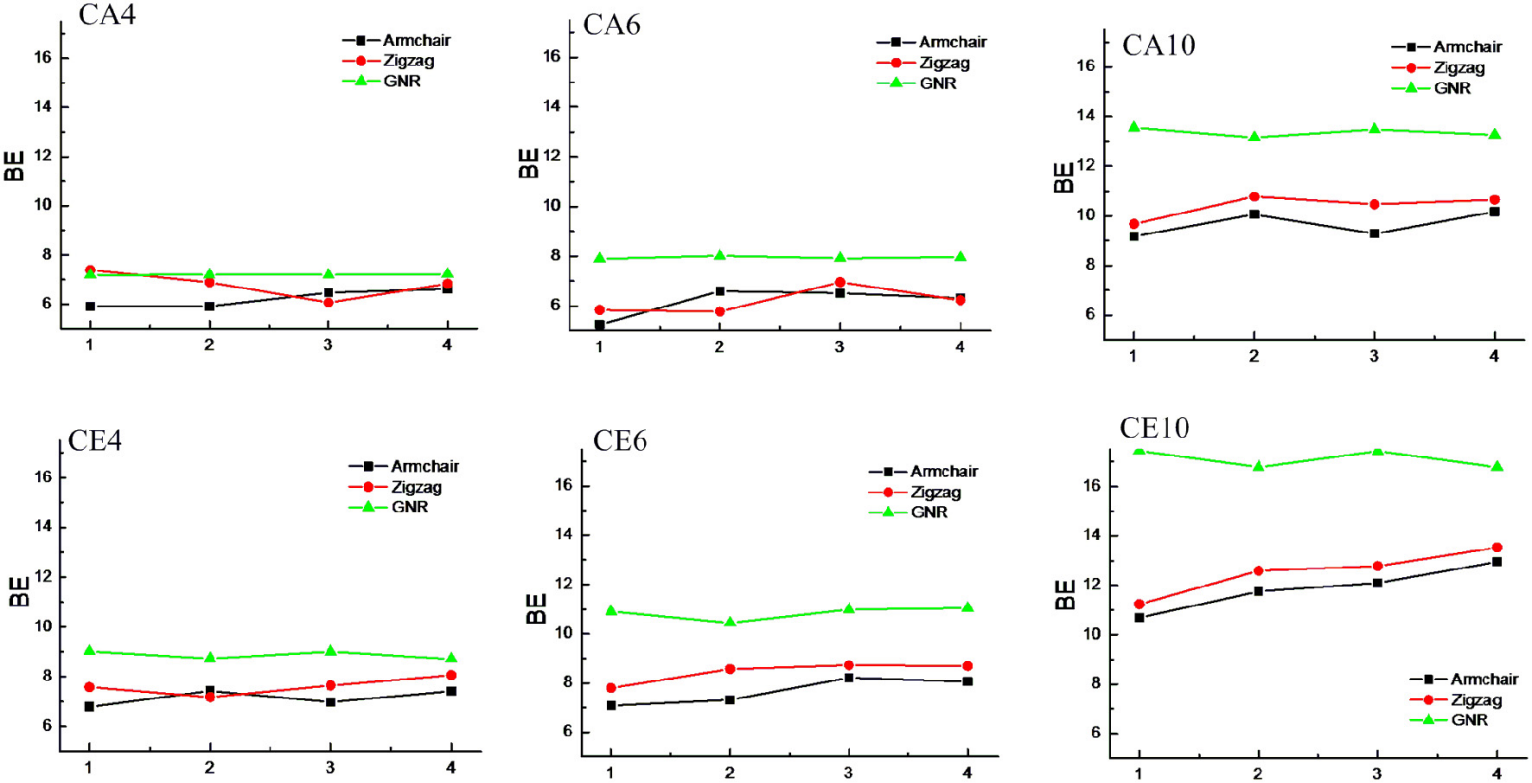
**Effect of curvature and chirality on the binding affinity of cyclic hydrocarbons with CNSs**.

CNTs have been known to exist in different chirality such as armchair CNTs and zigzag CNTs. As the CNTs are considered to be the rolled form of graphene sheet in one dimension, the chirality of the CNTs is envisaged by the rolling of graphene sheet in different orientations (Charlier, 2002). Based on the orientation of the tube axis with respect to the plane of the graphene sheet the structure of a CNT can be specified by chiral indices (n,m). Armchair CNTs which exhibit metallic behavior are represented as CNT (n,n) and the zigzag nanotubes are semiconductors which are defined as CNT (n,0). In order to explore the effect of CNTs chirality on the binding affinity toward various hydrocarbons, we have considered armchair and zigzag CNT models of comparable diameters. A systematic comparison of the BE of the armchair CNTs, zigzag CNTs and GNRs have been given in Figures 8, 9 for the acyclic and cyclic hydrocarbons, respectively. It is apparent from the figures that, virtually there is no change in the BE as the chirality of the CNTs varies.

While the studies on the interaction of planar π–systems have been explored in the literature, several studies on the curved π–systems have also been gradually forthcoming in recent years. To discuss the effect of curvature on the BE, both curved and planar model systems have been considered as shown in Figure 2. In order to represent the planar π–systems, we have considered GNR1, GNR2, GNR3, and GNR4 model systems. The models used to mimic the armchair CNTs such as CNT (4,4), (5,5), CNT (6,6), and CNT (7,7) are the rolled form of the graphene nanoribbons GNR1, GNR2, GNR3, and GNR4, respectively. Hence the effect of curvature can be studied by comparing the BE of these model systems. Let us revisit Tables 2, 3, where the BE of all the hydrocarbon complexes have been given. It is clear from the table that the BE of the planar GNRs are higher than that of the corresponding curved π–systems i.e., CNTs. It is also interesting to note that difference in the BE of the planar and curved systems increases as the size of the hydrocarbon increases. Figures 8, 9 have also reinforced the fact that the flat graphene shows strong binding affinity than the curved CNTs. Thus, the preceding analysis essentially quantifies the impact of curvature and chirality of the π–π and CH···π interaction of hydrocarbons with the CNSs.

### Comparison with the available experimental results

In this section, we have compared the available experimental observations with our computational results and the result is very encouraging. The foregoing discussion on the BE of the CNSs-hydrocarbon molecules clearly depicted the contrasting binding nature of CNSs toward the acyclic and cyclic hydrocarbons. In the case of acyclic hydrocarbons, the binding affinity of A*n* has been found to be greater than E*n*, while for cyclic hydrocarbons, CE*n* tends to bind more strongly than the corresponding CAn. The BE increases as the size of the hydrocarbon increases in all the cases. In addition, the acyclic hydrocarbons show stronger binding affinity than the cyclic hydrocarbons toward the CNSs.

Interestingly, the results obtained from our computational study have found to be in good agreement with the earlier experimental results on CNSs. Díaz et al. studied the adsorption of hydrocarbons by inverse gas chromatography and reported that the adsorption energy increases as the size of the hydrocarbon increases (Díaz et al., 2007). Several experimental and computational studies showed the preference of CNTs toward ethane than ethylene (Cruz and Müller, 2009; Albesa et al., 2011; Xingling et al., 2013). Sumanasekera et al. reported from their experimental studies that the adsorption energy of SWNT complexes with benzene is 9.42 kJ/mol and that of cyclohexane is 7.59 kJ/mol. They have shown systematically that the BE decreases as we go from CE*n* to CA*n* (Sumanasekera et al., 2002). The free energy of adsorption of benzene over CNTs have been found to be more than that of the free energy of adsorption of cyclohexane in an experimental study (Díaz et al., 2007). It has also been shown from the same study that the free energy of adsorption of acyclic hydrocarbon is higher than that of the cyclic counterpart. Agnihotri et al. showed from their experimental study on the electric arc SWCNT sample that the adsorption energy of hexane is higher than the cyclohexane (Agnihotri et al., 2005).

### AIM analysis

We have employed AIM analysis in order to understand the noncovalent interaction between the hydrocarbons and CNSs. We have considered CNT(4,4), CNT(8,0), and GNR1 as representative cases to study the topology of electron density of the complexes of armchair CNTs, zigzag CNTs and GNRs, respectively. The sign of the Laplacian of the density, ∇^2^ρ, is a broadly used tool to differentiate shared bonds and closed shell bonds. A closed shell bond can be either a noncovalent interaction or a polar-covalent bond like organo-metallic bonds (Cortés-Guzmán and Bader, 2005; Foroutan-Nejad et al., 2014). In general, if the sign of the Laplacian values are positive, then they are considered to be closed shell bonds (Higashibayashi et al., 2013). For the CNS-hydrocarbon complexes, it has been observed that the Laplacian values are positive and that confirms the existence of closed shell bonds and noncovalent interactions in this case. As several LCPs have been observed in between the CNSs and the hydrocarbons, we have considered the sum of the electron density values at various LCPs in the stacked regions (Table 4). It is evident from the table that the sum of the electron densities increases as the size of the systems increases. Besides, the sum of the electron densities in the complexes of saturated hydrocarbons has been found to be higher than their unsaturated counterparts in most of the cases.

**Table 4:** **Sum of the electron densities at the LCPs of CNSs-hydrocarbon complexes in a.u. obtained by AIM analysis**.

	**A*n***	**E*n***		**CA*n***	**CE*n***
**CNT**					
*n* = 2	0.0225	0.0142	*n* = 4	0.0302	0.0273
*n* = 4	0.0258	0.0269	*n* = 6	0.0303	0.0246
*n* = 6	0.0535	0.0265	*n* = 10	0.0504	0.0333
**Z*N*T**					
*n* = 2	0.0197	0.0125	*n* = 4	0.0382	0.0260
*n* = 4	0.0324	0.0244	*n* = 6	0.0360	0.0261
*n* = 6	0.0505	0.0145	*n* = 10	0.0421	0.0390
**G*N*R**					
*n* = 2	0.0129	0.0148	*n* = 4	0.0189	0.0307
*n* = 4	0.0294	0.0224	*n* = 6	0.0270	0.0275
*n* = 6	0.0512	0.0398	*n* = 10	0.0410	0.0347

**Figure 10:**
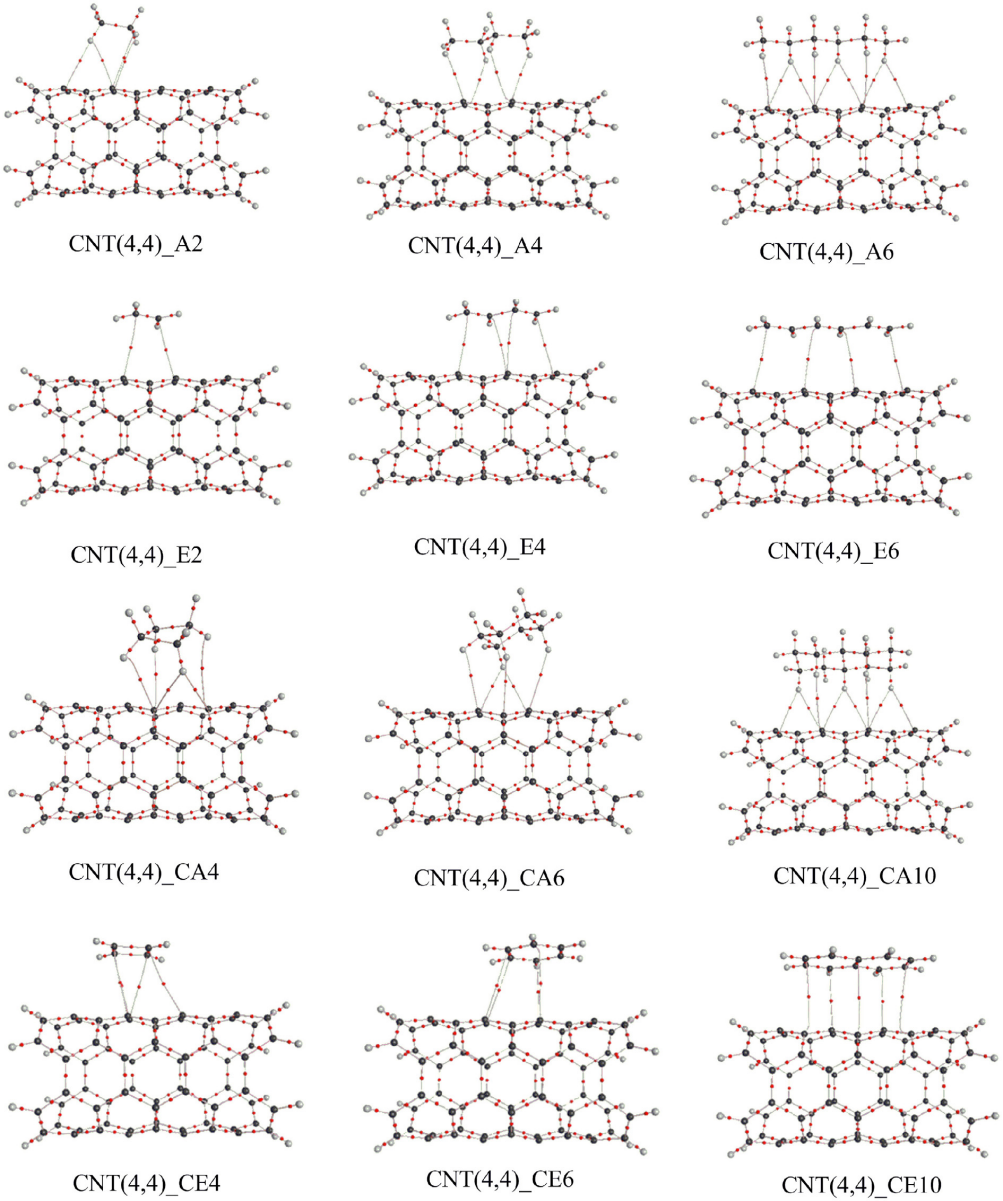
Atomic positions and LCPs of CNT complexes with hydrocarbons obtained at M06-2X/6-31G^*^ level.

To get further understanding, the topology of electron density of the complexes has been given in the Figure 10. It is evident from the figure that there are two different LCPs occurred in between the hydrocarbon and the CNSs such as C···C and CH···C. It is also interesting to note that, in the case of saturated hydrocarbon complexes such as A*n* and CA*n* the LCPs are obtained between *H* of the hydrocarbon and *C* of the CNSs. However, for unsaturated hydrocarbons, E*n* and CE*n*, the LCPs are obtained between *C* of the hydrocarbon and *C* of the CNSs. Thus, the contrasting nature of the LCPs in the complexes of saturated and unsaturated hydrocarbon toward the CNSs is clearly brought out by the AIM analysis.

## Conclusions

In this work, a systematic study has been done to understand the interaction of saturated and unsaturated hydrocarbons with the CNSs. The saturated molecules show stronger binding affinity in acyclic hydrocarbons whereas the unsaturated molecules exhibit stronger binding affinity in cyclic hydrocarbons. It has been observed that the BE of the CNSs-hydrocarbon complexes increases as the size of the hydrocarbon increases. It is interesting to note that acyclic hydrocarbons show stronger binding affinity than the cyclic hydrocarbons toward the CNSs. Our results indicate that planar graphene exhibit stronger binding affinity toward the hydrocarbons when compared to the curved CNTs. The results obtained from our computational study have been found to be in good agreement with the earlier experimental results on CNSs. The theory of AIM provides a rational basis for differentiating the complexes of saturated and unsaturated hydrocarbons. We hope that our results would be very useful in understanding the various applications of CNSs.

### Conflict of interest statement

The authors declare that the research was conducted in the absence of any commercial or financial relationships that could be construed as a potential conflict of interest.

## Supplementary material

Optimized structures of possible orientations of hydrocarbons on CNSs; Optimized geometries of the complexes considered; AIM molecular graph of zigzag CNTs and GNR.
