# Supplementary material for: Saturated vs. unsaturated hydrocarbon interactions with carbon nanostructures
Source: Front Chem. 2014 Sep 3;2:75. doi: 10.3389/fchem.2014.00075 (PMC4153313; doi:10.3389/fchem.2014.00075)
Supplement: Supplementary file 1 [file DataSheet1.PDF]

# Saturated versus unsaturated hydrocarbon interactions with carbon nanostructures

Deivasigamani Umadevi and G. Narahari Sastry*
Centre for Molecular Modeling, CSIR - Indian Institute of Chemical Technology, Hyderabad - 500 607, INDIA, Phone- +91 40 27193016, E-mail: gnsastry@gmail.com

## Supplementary Material


| S.No | Table | Page No |
|---|---|---|
| **S1** | The shortest distance between CNSs and the hydrocarbons (r in Å) between the CNSs-acyclic hydrocarbon complexes. | 3 |
| **S2** | The shortest distance between CNSs and the hydrocarbons (r in Å) between the CNSs-cyclic hydrocarbon complexes. | 4 |
| **S3** | The electron charge density (ρ in a.u.) and the Laplacian of the electron density ($\nabla^2\rho$ in a.u.) at the bond critical points of the armchair CNTs-acyclic hydrocarbon complexes using the wave functions obtained from the M06-2X/6-31G* calculation. | 5 |
| **S4** | The electron charge density (ρ in a.u.) and the Laplacian of the electron density ($\nabla^2\rho$ in a.u.) at the bond critical points of the armchair CNTs-cyclic hydrocarbon complexes using the wave functions obtained from the M06-2X/6-31G* calculation. | 6 |
| **S5** | The electron charge density (ρ in a.u.) and the Laplacian of the electron density ($\nabla^2\rho$ in a.u.) at the bond critical points of the zigzag CNTs-acyclic hydrocarbon complexes using the wave functions obtained from the M06-2X/6-31G* calculation. | 7 |
| **S6** | The electron charge density (ρ in a.u.) and the Laplacian of the electron density ($\nabla^2\rho$ in a.u.) at the bond critical points of the zigzag CNTs-cyclic hydrocarbon complexes using the wave functions obtained from the M06-2X/6-31G* calculation | 8 |

| S.No | Figure | Page No |
|---|---|---|
| **S1** | Possible orientations (S and T) of the unsaturated hydrocarbons on CNSs and their binding energies (kcal/mol). | 9 |
| **S2** | Optimized structures of possible orientations of the A*n* complexes with CNT(4,4) and the corresponding binding energies (kcal/mol) at M06-2X/6-311G**//ONIOM(M06-2X/6-31G*:AM1) level | 10 |
| **S3** | Optimized structures of possible orientations of the En complexes with CNT(4,4) and the corresponding binding energies (kcal/mol) at M06-2X/6-311G**//ONIOM(M06-2X/6-31G*:AM1) level. | 11 |

| | | |
|---|---|---|
| **S4** | Optimized structures of possible orientations of the CA*n* complexes with CNT(4,4) and the corresponding binding energies (kcal/mol) at M06-2X/6-311G**//ONIOM(M06-2X/6-31G*:AM1) level | 12 |
| **S5** | Optimized structures of possible orientations of the CA*n* complexes with CNT(4,4) and the corresponding binding energies (kcal/mol) at M06-2X/6-311G**//ONIOM(M06-2X/6-31G*:AM1) level | 13 |
| **S6** | Optimized geometries of the complexes of armchair CNTs with A*n* at ONIOM(M06-2X/6-31G*:AM1) level and the nearest distance (Å) between them. | 14 |
| **S7** | Optimized geometries of the complexes of armchair CNTs with E*n* at ONIOM(M06-2X/6-31G*:AM1) level and the nearest distance (Å) between them | 15 |
| **S8** | Optimized geometries of the complexes of armchair CNTs with CA*n* at ONIOM(M06-2X/6-31G*:AM1) level and the nearest distance (Å) between them | 16 |
| **S9** | Optimized geometries of the complexes of armchair CNTs with CE*n* at ONIOM(M06-2X/6-31G*:AM1) level and the nearest distance (Å) between them | 17 |
| **S10** | Optimized geometries of the complexes of zigzag CNTs with A*n* at ONIOM(M06-2X/6-31G*:AM1) level and the nearest distance (Å) between them | 18 |
| **S11** | Optimized geometries of the complexes of zigzag CNTs with E*n* at ONIOM(M06-2X/6-31G*:AM1) level and the nearest distance (Å) between them | 19 |
| **S12** | Optimized geometries of the complexes of zigzag CNTs with CA*n* at ONIOM(M06-2X/6-31G*:AM1) level and the nearest distance (Å) between them | 20 |
| **S13** | Optimized geometries of the complexes of zigzag CNTs with CE*n* at ONIOM(M06-2X/6-31G*:AM1) level and the nearest distance (Å) between them | 21 |
| **S14** | Optimized geometries of the complexes of GNRs with A*n* at ONIOM(M06-2X/6-31G*:AM1) level and the nearest distance (Å) between them | 22 |
| **S15** | Optimized geometries of the complexes of GNRs with E*n* at ONIOM(M06-2X/6-31G*:AM1) level and the nearest distance (Å) between them | 23 |
| **S16** | Optimized geometries of the complexes of GNRs with CA*n* at ONIOM(M06-2X/6-31G*:AM1) level and the nearest distance (Å) between them | 24 |
| **S17** | Optimized geometries of the complexes of GNRs with CE*n* at ONIOM(M06-2X/6-31G*:AM1) level and the nearest distance (Å) between them | 25 |
| **S18** | Atomic positions and critical points of CNT complexes with hydrocarbons obtained at M06-2X/6-31G* level. BCPs are represented by red color dots | 26 |
| **S19** | Atomic positions and critical points of CNT complexes with hydrocarbons obtained at M06-2X/6-31G* level. BCPs are represented by red color dots | 27 |

**Table S1**: The shortest distance between CNSs and the hydrocarbons (r in Å) between the CNSs-acyclic hydrocarbon complexes

| CNT | | | ZNT | | | GRAPHENE | | |
|---|---|---|---|---|---|---|---|---|
| **CNT(4,4)** | A*n* | E*n* | **CNT(8,0)** | A*n* | E*n* | **GNR1** | A*n* | E*n* |
| ***n*=2** | 2.576 | 3.027 | ***n*=2** | 2.379 | 3.384 | ***n*=2** | 2.557 | 3.134 |
| ***n*=4** | 2.566 | 3.032 | ***n*=4** | 2.623 | 3.028 | ***n*=4** | 2.514 | 2.971 |
| ***n*=6** | 2.337 | 3.231 | ***n*=6** | 2.033 | 3.167 | ***n*=6** | 2.582 | 3.187 |
| | | | | | | | | |
| **CNT (5,5)** | | | **CNT(10,0)** | | | **GNR2** | | |
| ***n*=2** | 2.568 | 3.156 | ***n*=2** | 2.534 | 3.303 | ***n*=2** | 2.546 | 3.073 |
| ***n*=4** | 2.578 | 3.068 | ***n*=4** | 2.375 | 3.045 | ***n*=4** | 2.511 | 2.981 |
| ***n*=6** | 2.354 | 3.220 | ***n*=6** | 2.254 | 3.126 | ***n*=6** | 2.573 | 3.175 |
| | | | | | | | | |
| **CNT (6,6)** | | | **CNT(12,0)** | | | **GNR3** | | |
| ***n*=2** | 2.551 | 3.145 | ***n*=2** | 2.559 | 3.332 | ***n*=2** | 2.557 | 3.124 |
| ***n*=4** | 2.592 | 3.026 | ***n*=4** | 2.375 | 3.042 | ***n*=4** | 2.514 | 2.971 |
| ***n*=6** | 2.332 | 3.188 | ***n*=6** | 2.331 | 3.154 | ***n*=6** | 2.579 | 3.187 |
| | | | | | | | | |
| **CNT (7,7)** | | | **CNT(14,0)** | | | **GNR4** | | |
| ***n*=2** | 2.565 | 3.139 | ***n*=2** | 2.568 | 3.302 | ***n*=2** | 2.551 | 3.070 |
| ***n*=4** | 2.597 | 3.049 | ***n*=4** | 2.366 | 3.128 | ***n*=4** | 2.505 | 2.972 |
| ***n*=6** | 2.334 | 3.153 | ***n*=6** | 2.294 | 3.138 | ***n*=6** | 2.569 | 3.176 |

**Table S2:** The shortest distance between CNSs and the hydrocarbons (r in Å) between the CNSs-cyclic hydrocarbon complexes

| CNT | | | ZNT | | | GRAPHENE | | |
|---|---|---|---|---|---|---|---|---|
| | CA*n* | CE*n* | | CA*n* | CE*n* | | CA*n* | CE*n* |
| **CNT(4,4)** | | | **CNT(8,0)** | | | **GNR1** | | |
| ***n*=4** | 2.430 | 3.128 | ***n*=4** | 2.565 | 2.928 | ***n*=4** | 2.392 | 3.122 |
| ***n*=6** | 2.415 | 3.209 | ***n*=6** | 2.514 | 3.253 | ***n*=6** | 2.347 | 3.206 |
| ***n*=10** | 2.442 | 3.104 | ***n*=10** | 2.338 | 2.531 | ***n*=10** | 2.384 | 3.304 |
| | | | | | | | | |
| **CNT (5,5)** | | | **CNT(10,0)** | | | **GNR2** | | |
| ***n*=4** | 2.452 | 3.131 | ***n*=4** | 2.668 | 3.088 | ***n*=4** | 2.379 | 3.105 |
| ***n*=6** | 2.443 | 3.117 | ***n*=6** | 2.441 | 3.215 | ***n*=6** | 2.323 | 3.236 |
| ***n*=10** | 2.482 | 3.073 | ***n*=10** | 2.335 | 3.205 | ***n*=10** | 2.355 | 3.340 |
| | | | | | | | | |
| **CNT (6,6)** | | | **CNT(12,0)** | | | **GNR3** | | |
| ***n*=4** | 2.458 | 3.126 | ***n*=4** | 2.490 | 3.128 | ***n*=4** | 2.379 | 3.113 |
| ***n*=6** | 2.429 | 3.112 | ***n*=6** | 2.433 | 3.055 | ***n*=6** | 2.352 | 3.198 |
| ***n*=10** | 2.444 | 3.015 | ***n*=10** | 2.346 | 3.222 | ***n*=10** | 2.392 | 3.292 |
| | | | | | | | | |
| **CNT (7,7)** | | | **CNT(14,0)** | | | **GNR4** | | |
| ***n*=4** | 2.426 | 3.125 | ***n*=4** | 2.690 | 3.098 | ***n*=4** | 2.373 | 3.105 |
| ***n*=6** | 2.443 | 3.126 | ***n*=6** | 2.623 | 3.274 | ***n*=6** | 2.290 | 3.191 |
| ***n*=10** | 2.474 | 3.087 | ***n*=10** | 2.342 | 3.224 | ***n*=10** | 2.363 | 3.292 |

**Table S3:** The electron charge density (ρ in a.u.) and the Laplacian of the electron density ($\nabla^2\rho$ in a.u.) at the bond critical points of the armchair CNTs-acyclic hydrocarbon complexes using the wave functions obtained from the M06-2X/6-31G* calculation

| **A** | **ρ** | $\nabla^2\rho$ | **E** | **ρ** | $\nabla^2\rho$ |
|---|---|---|---|---|---|
| A2 | 0.0053 | 0.0042 | E2 | 0.0073 | 0.0073 |
| | 0.0059 | 0.0050 | | 0.0069 | 0.0069 |
| | 0.0057 | 0.0047 | | | |
| | 0.0057 | 0.0047 | | | |
| | | | | | |
| A4 | 0.0066 | 0.0054 | E4 | 0.0076 | 0.0053 |
| | 0.0063 | 0.0051 | | 0.0066 | 0.0050 |
| | 0.0063 | 0.0051 | | 0.0066 | 0.0049 |
| | 0.0066 | 0.0054 | | 0.0060 | 0.0040 |
| | | | | | |
| A5 | 0.0057 | 0.0046 | E6 | 0.0066 | 0.0044 |
| | 0.0065 | 0.0052 | | 0.0067 | 0.0049 |
| | 0.0061 | 0.0051 | | 0.0067 | 0.0049 |
| | 0.0060 | 0.0048 | | 0.0066 | 0.0044 |
| | 0.0058 | 0.0046 | | | |
| | 0.0059 | 0.0047 | | | |
| | 0.0064 | 0.0050 | | | |
| | 0.0053 | 0.0043 | | | |
| | 0.0059 | 0.0047 | | | |

**Table S4:** The electron charge density (ρ in a.u.) and the Laplacian of the electron density ($\nabla^2\rho$ in a.u.) at the bond critical points of the armchair CNTs-cyclic hydrocarbon complexes using the wave functions obtained from the M06-2X/6-31G* calculation

| **CA** | ρ | $\nabla^2\rho$ | **CE** | ρ | $\nabla^2\rho$ |
|---|---|---|---|---|---|
| CA4 | 0.0038 | 0.0030 | CE4 | 0.0072 | 0.0056 |
| | 0.0067 | 0.0056 | | 0.0073 | 0.0057 |
| | 0.0071 | 0.0059 | | 0.0074 | 0.0057 |
| | 0.0071 | 0.0060 | | 0.0054 | 0.0038 |
| | 0.0054 | 0.0045 | | | |
| CA6 | 0.0047 | 0.0037 | CE6 | 0.0057 | 0.0042 |
| | 0.0074 | 0.0058 | | 0.0056 | 0.0041 |
| | 0.0074 | 0.0061 | | 0.0065 | 0.0046 |
| | 0.0071 | 0.0057 | | 0.0069 | 0.0049 |
| | 0.0037 | 0.0027 | | | |
| CA10 | 0.0073 | 0.0058 | CE10 | 0.0062 | 0.0044 |
| | 0.0061 | 0.0051 | | 0.0076 | 0.0052 |
| | 0.0049 | 0.0035 | | 0.0056 | 0.0041 |
| | 0.0070 | 0.0054 | | 0.0076 | 0.0052 |
| | 0.0068 | 0.0052 | | 0.0064 | 0.0044 |
| | 0.0044 | 0.0032 | | | |
| | 0.0065 | 0.0053 | | | |
| | 0.0074 | 0.0059 | | | |

**Table S5:** The electron charge density (ρ in a.u.) and the Laplacian of the electron density ($\nabla^2\rho$ in a.u.) at the bond critical points of the zigzag CNTs-acyclic hydrocarbon complexes using the wave functions obtained from the M06-2X/6-31G* calculation

| **A** | ρ | $\nabla^2\rho$ | **E** | ρ | $\nabla^2\rho$ |
|---|---|---|---|---|---|
| A2 | 0.0056 | 0.0044 | E2 | 0.0063 | 0.0045 |
| | 0.0073 | 0.0061 | | 0.0063 | 0.0044 |
| | 0.0067 | 0.0057 | | | |
| A4 | 0.0059 | 0.0049 | E4 | 0.0054 | 0.0037 |
| | 0.0067 | 0.0055 | | 0.0070 | 0.0054 |
| | 0.0067 | 0.0056 | | 0.0070 | 0.0054 |
| | 0.0066 | 0.0054 | | 0.0050 | 0.0034 |
| | 0.0066 | 0.0054 | | | |
| A5 | 0.0069 | 0.0052 | E6 | 0.0073 | 0.0056 |
| | 0.0054 | 0.0044 | | 0.0072 | 0.0056 |
| | 0.0067 | 0.0055 | | | |
| | 0.0067 | 0.0057 | | | |
| | 0.0068 | 0.0057 | | | |
| | 0.0069 | 0.0057 | | | |
| | 0.0064 | 0.0050 | | | |
| | 0.0048 | 0.0038 | | | |

**Table S6:** The electron charge density (ρ in a.u.) and the Laplacian of the electron density ($\nabla^2\rho$ in a.u.) at the bond critical points of the zigzag CNTs-cyclic hydrocarbon complexes using the wave functions obtained from the M06-2X/6-31G* calculation

| **CA** | ρ | $\nabla^2\rho$ | **CE** | ρ | $\nabla^2\rho$ |
|---|---|---|---|---|---|
| CA4 | 0.0039 | 0.0031 | CE4 | 0.0068 | 0.00468 |
| | 0.0065 | 0.0055 | | 0.0092 | 0.00642 |
| | 0.0072 | 0.0062 | | 0.0100 | 0.00721 |
| | 0.0072 | 0.0062 | | | |
| | 0.0071 | 0.0062 | | | |
| | 0.0064 | 0.0053 | | | |
| CA6 | 0.0057 | 0.0044 | CE6 | 0.0083 | 0.00630 |
| | 0.0057 | 0.0048 | | 0.0069 | 0.00490 |
| | 0.0080 | 0.0064 | | 0.0068 | 0.00487 |
| | 0.0083 | 0.0069 | | 0.0040 | 0.00294 |
| | 0.0057 | 0.0045 | | | |
| | 0.0026 | 0.0020 | | | |
| CA10 | 0.0052 | 0.0043 | CE10 | 0.0067 | 0.00454 |
| | 0.0071 | 0.0056 | | 0.0055 | 0.00392 |
| | 0.0090 | 0.0075 | | 0.0078 | 0.00599 |
| | 0.0091 | 0.0075 | | 0.0063 | 0.00432 |
| | 0.0073 | 0.0059 | | 0.0070 | 0.00488 |
| | 0.0045 | 0.0034 | | 0.0059 | 0.00410 |

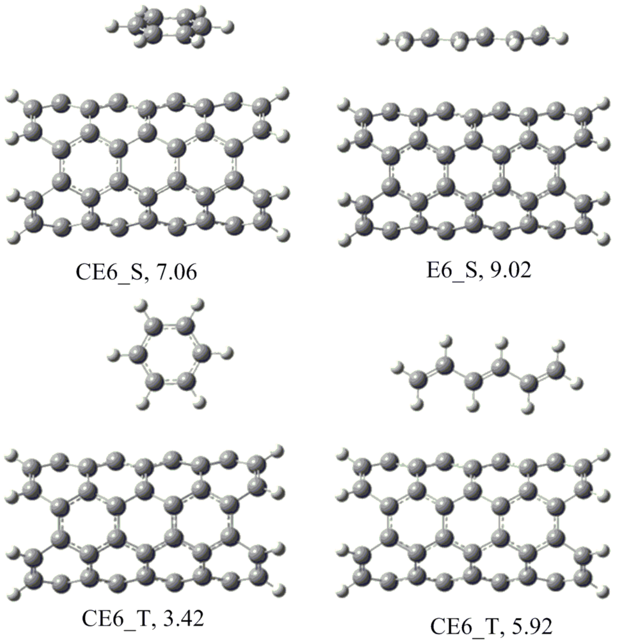


**Figure S1:** Possible orientations (S and T) of the unsaturated hydrocarbons on CNSs and their binding energies (kcal/mol).

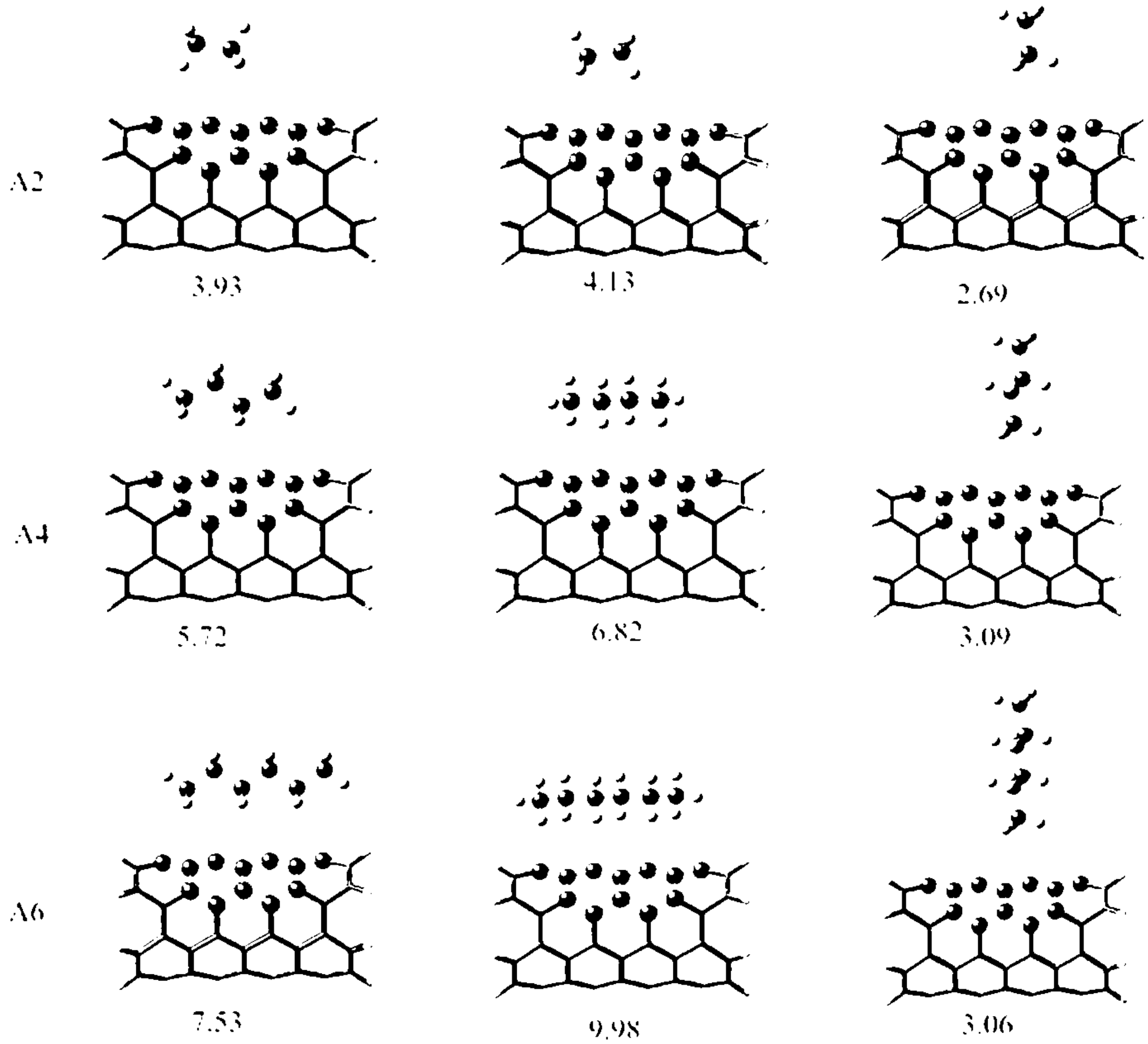


**Figure S2:** Optimized structures of possible orientations of the A*n* complexes with CNT(4,4) and the corresponding binding energies (kcal/mol) at M06-2X/6-311G**//ONIOM(M06-2X/6-31G*:AM1) level.

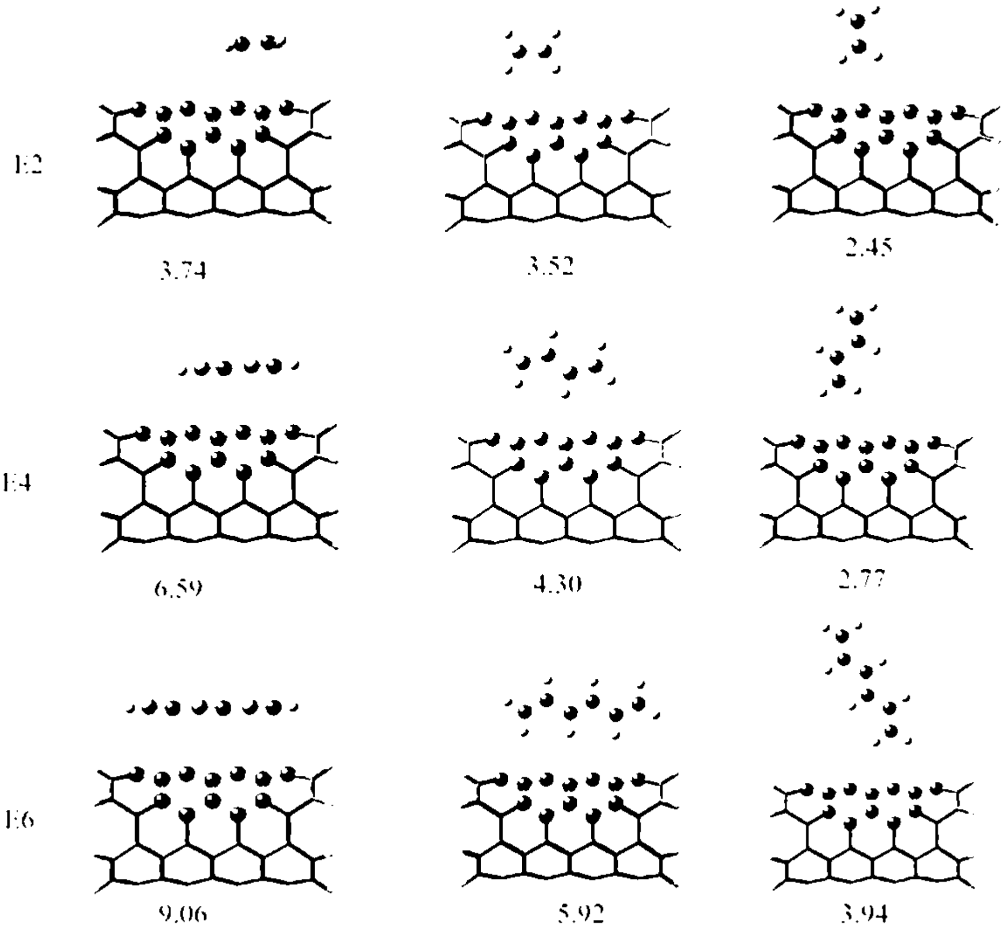


**Figure S3:** Optimized structures of possible orientations of the E*n* complexes with CNT(4,4) and the corresponding binding energies (kcal/mol) at M06-2X/6-311G**//ONIOM(M06-2X/6-31G*:AM1) level.

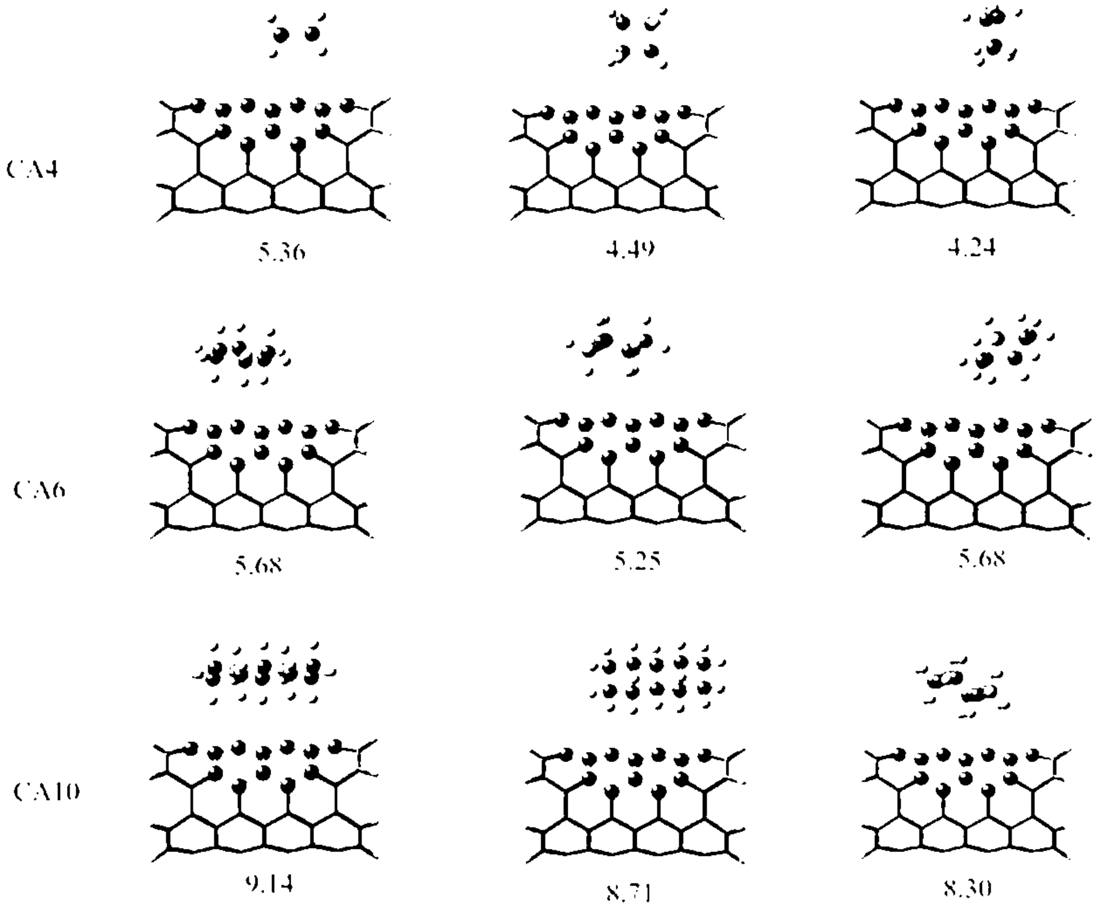


**Figure S4:** Optimized structures of possible orientations of the CA*n* complexes with CNT(4,4) and the corresponding binding energies (kcal/mol) at M06-2X/6-311G**//ONIOM(M06-2X/6-31G*:AM1) level.

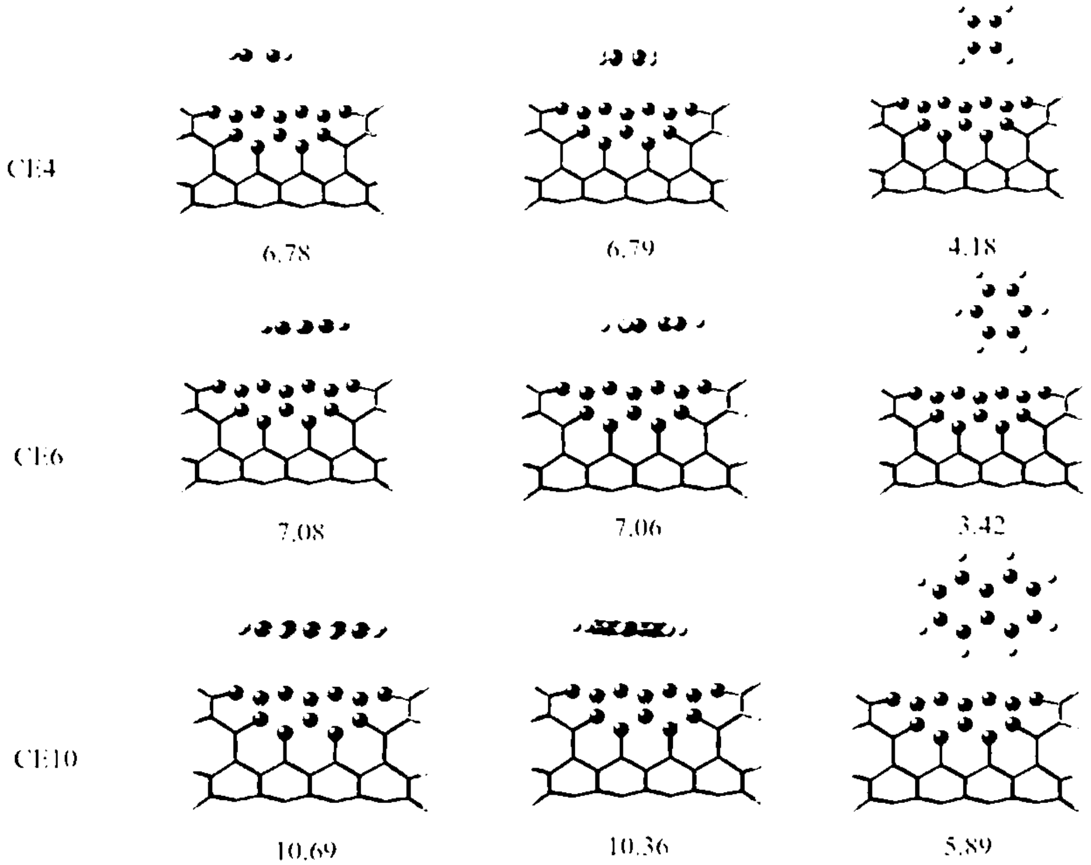


**Figure S5:** Optimized structures of possible orientations of the CA*n* complexes with CNT(4,4) and the corresponding binding energies (kcal/mol) at M06-2X/6-311G**//ONIOM(M06-2X/6-31G*:AM1) level.

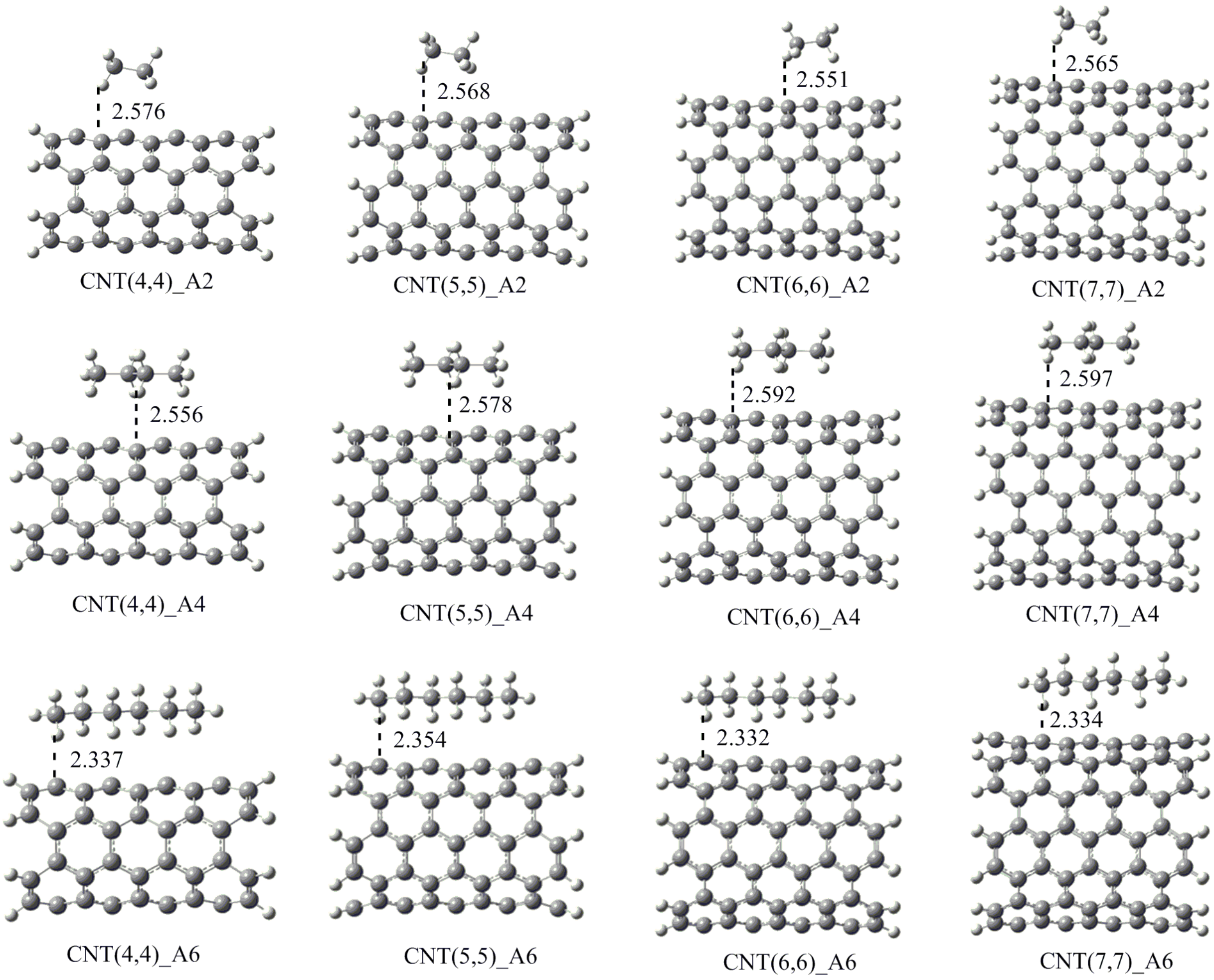


**Figure S6:** Optimized geometries of the complexes of armchair CNTs with A*n* at ONIOM(M06-2X/6-31G*:AM1) level and the nearest distance (Å) between them.

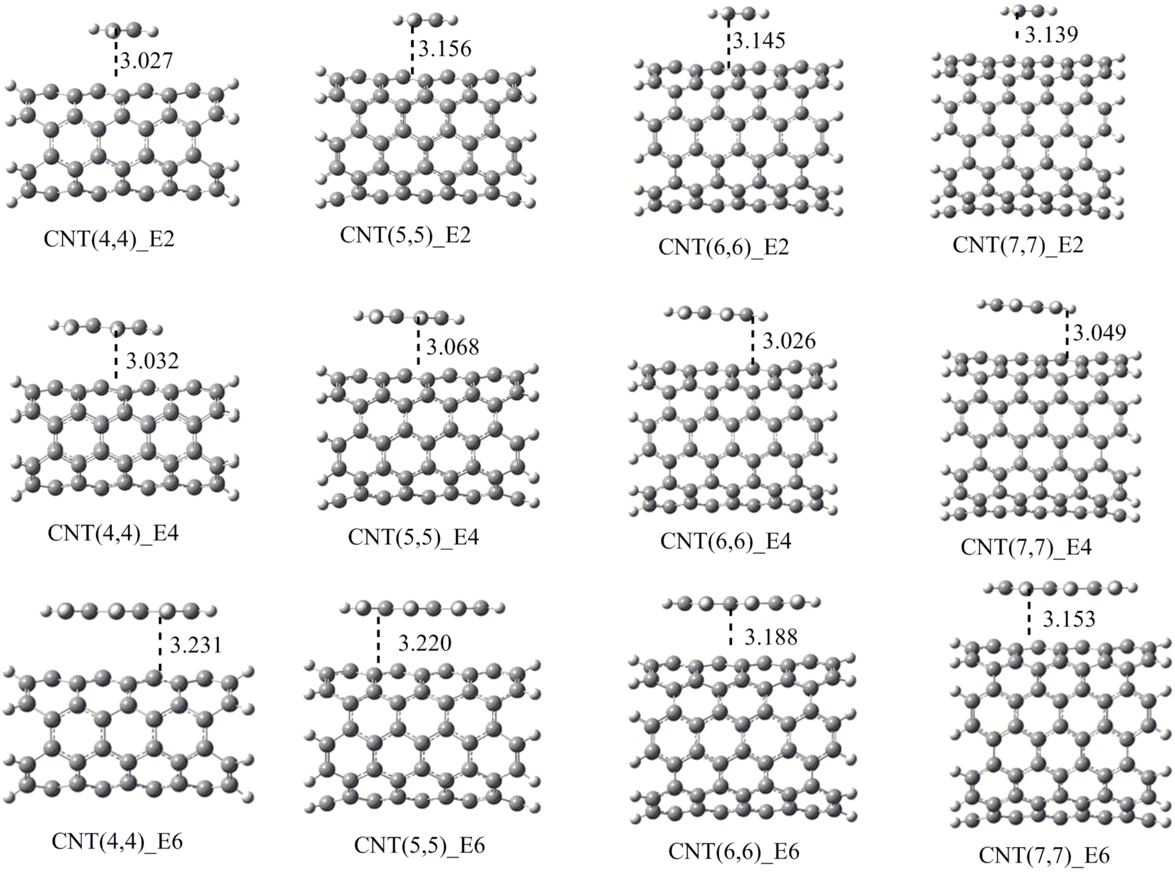


**Figure S7:** Optimized geometries of the complexes of armchair CNTs with E*n* at ONIOM(M06-2X/6-31G*:AM1) level and the nearest distance (Å) between them.

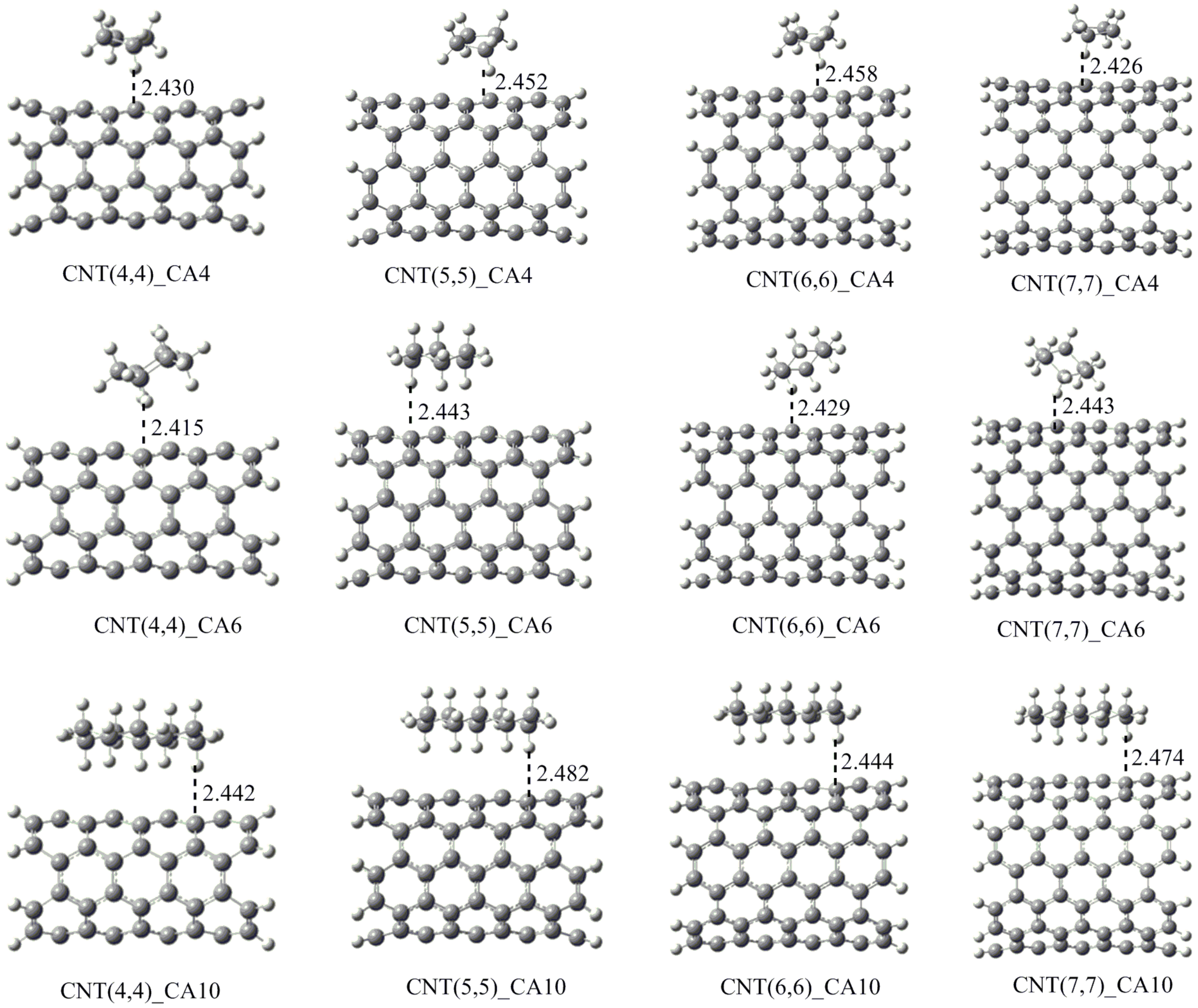


**Figure S8:** Optimized geometries of the complexes of armchair CNTs with CA*n* at ONIOM(M06-2X/6-31G*:AM1) level and the nearest distance (Å) between them.

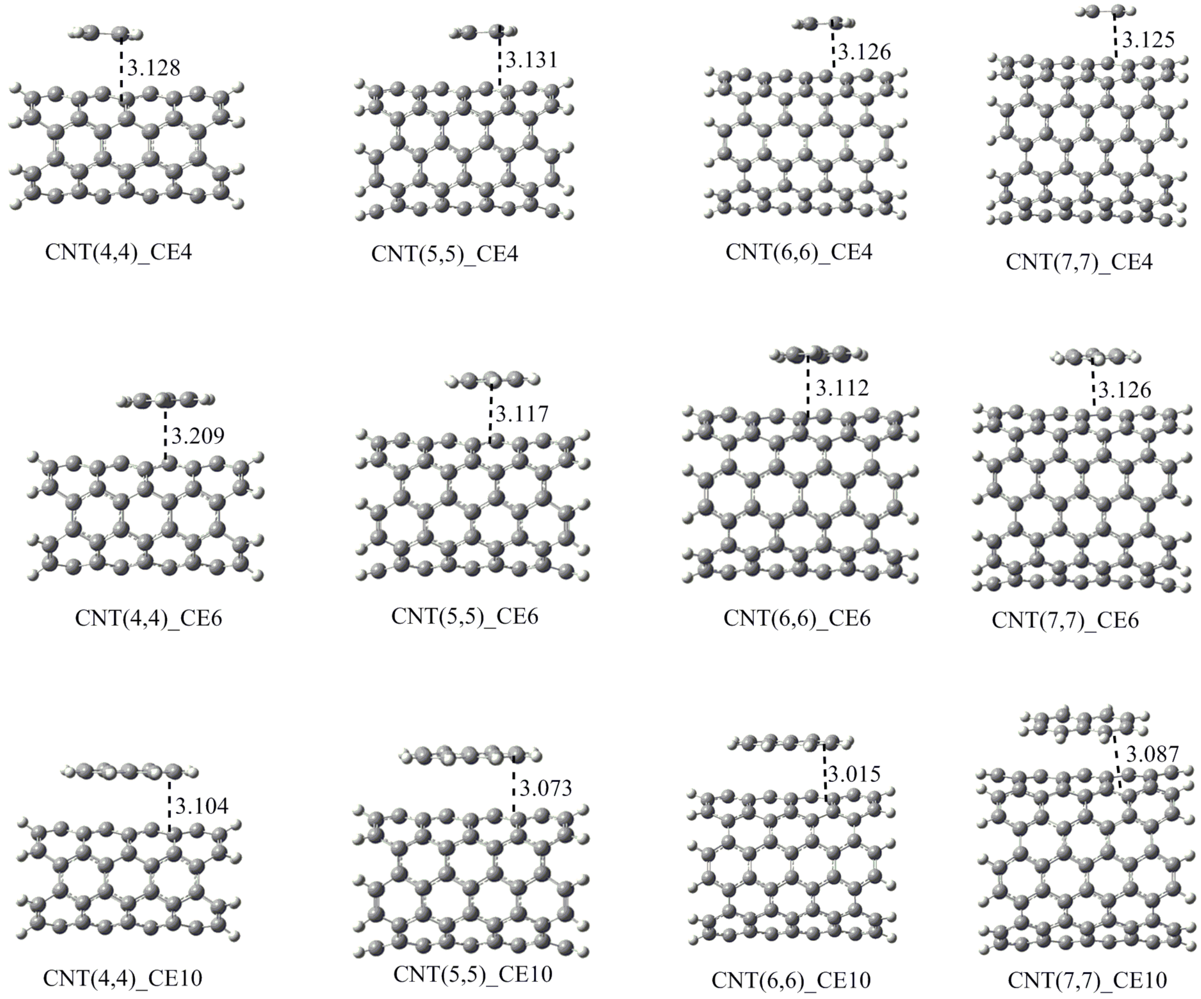


**Figure S9:** Optimized geometries of the complexes of armchair CNTs with CE*n* at ONIOM(M06-2X/6-31G*:AM1) level and the nearest distance (Å) between them.

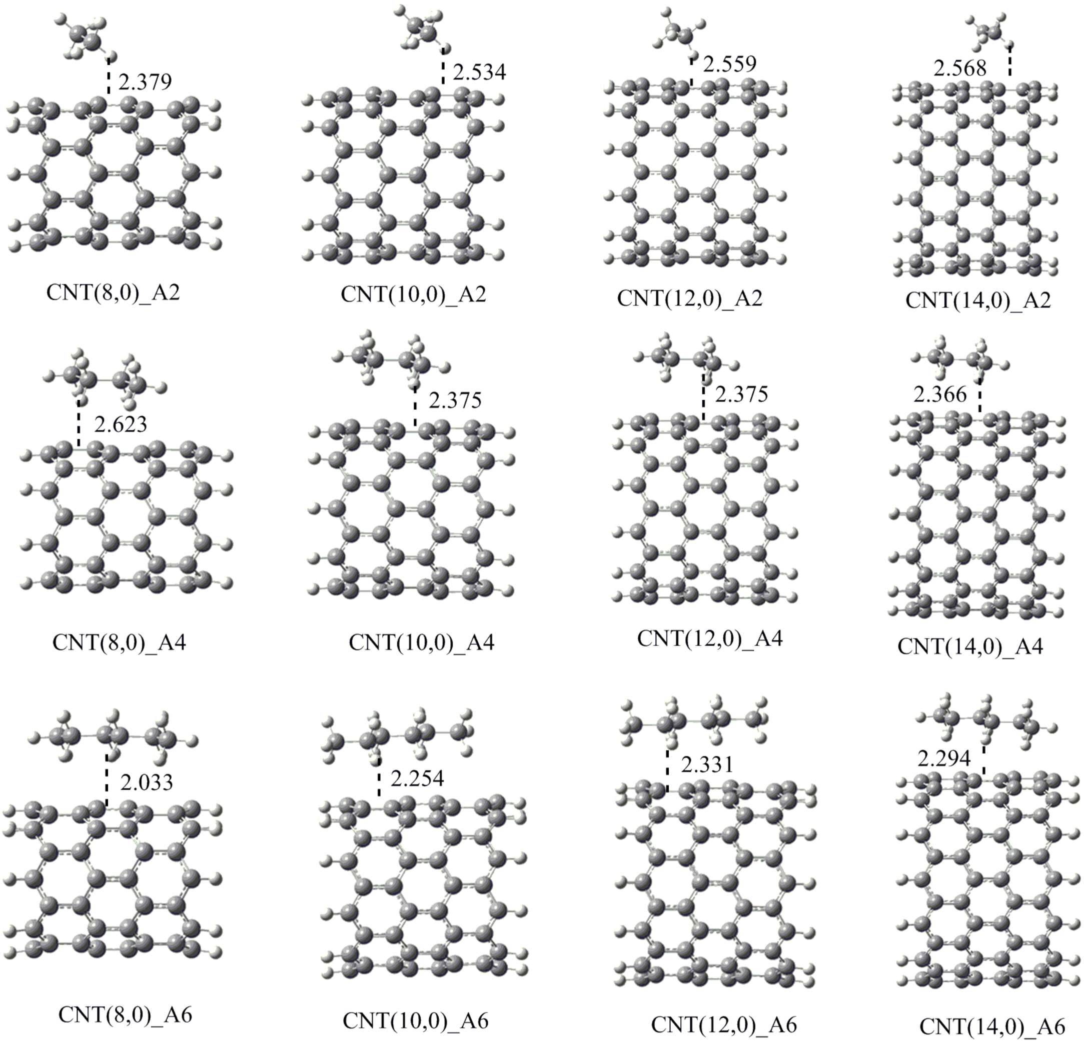


**Figure S10:** Optimized geometries of the complexes of zigzag CNTs with A*n* at ONIOM(M06-2X/6-31G*:AM1) level and the nearest distance (Å) between them.

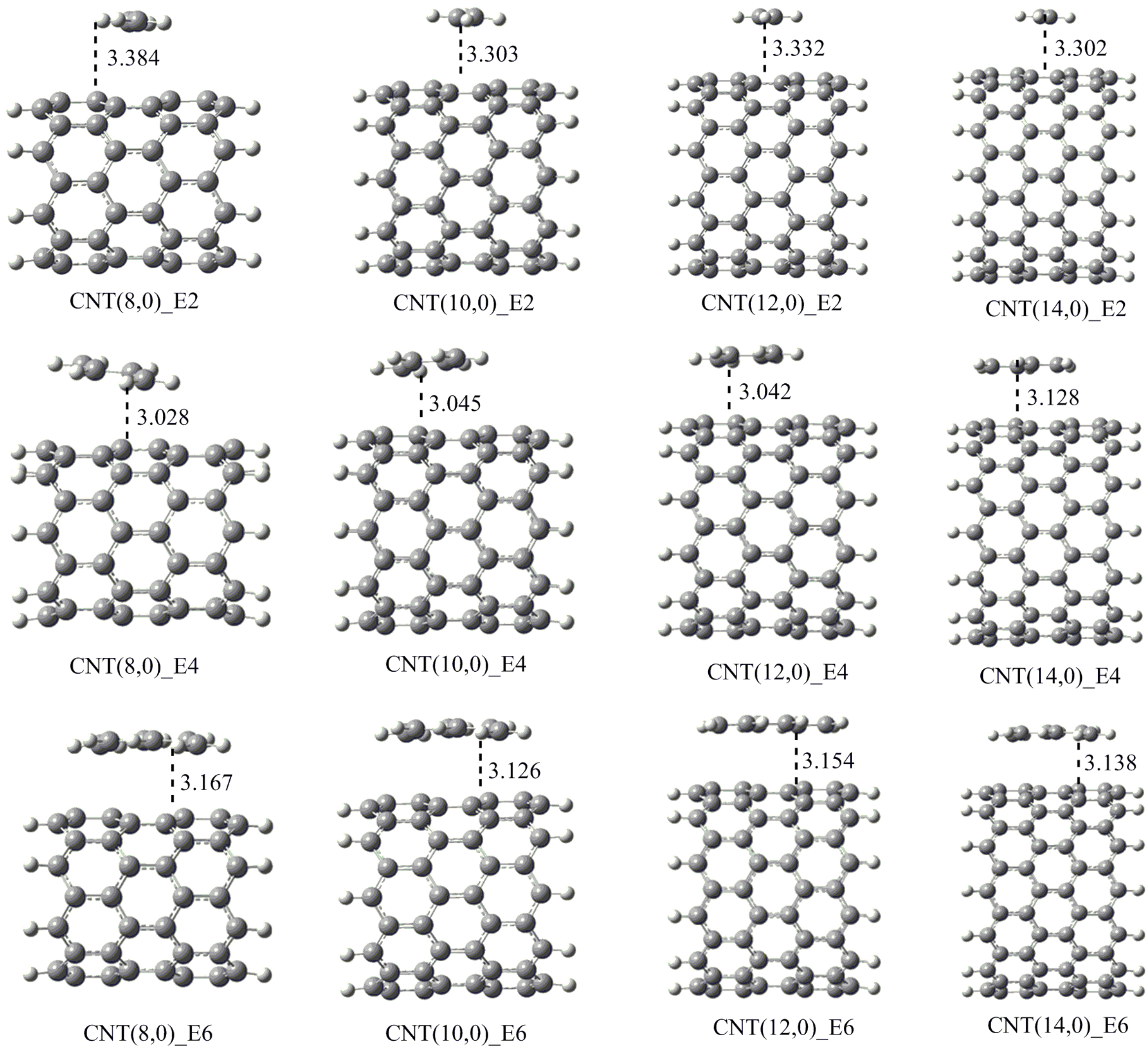


**Figure S11:** Optimized geometries of the complexes of zigzag CNTs with E*n* at ONIOM(M06-2X/6-31G*:AM1) level and the nearest distance (Å) between them.

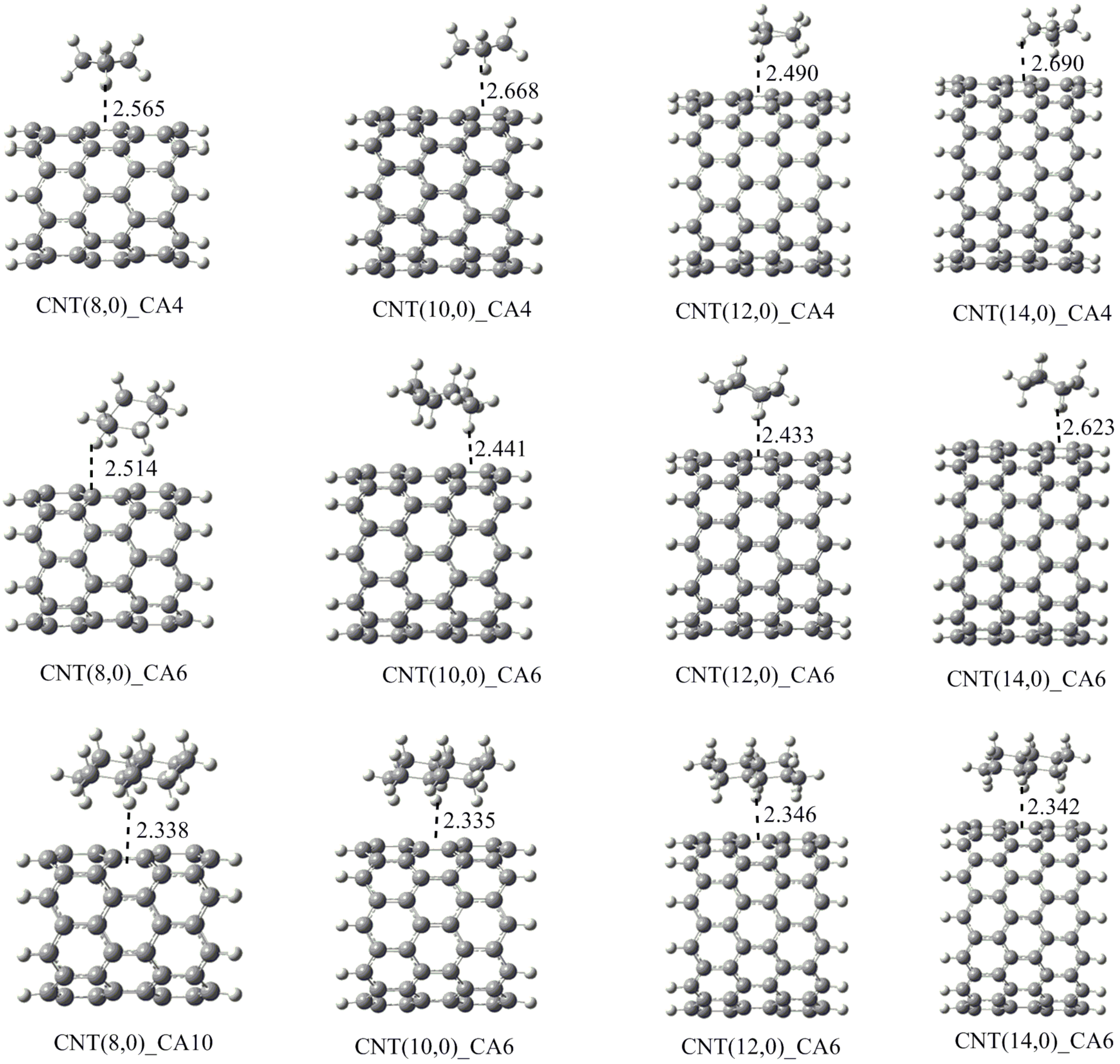


**Figure S12:** Optimized geometries of the complexes of zigzag CNTs with CA*n* at ONIOM(M06-2X/6-31G*:AM1) level and the nearest distance (Å) between them.

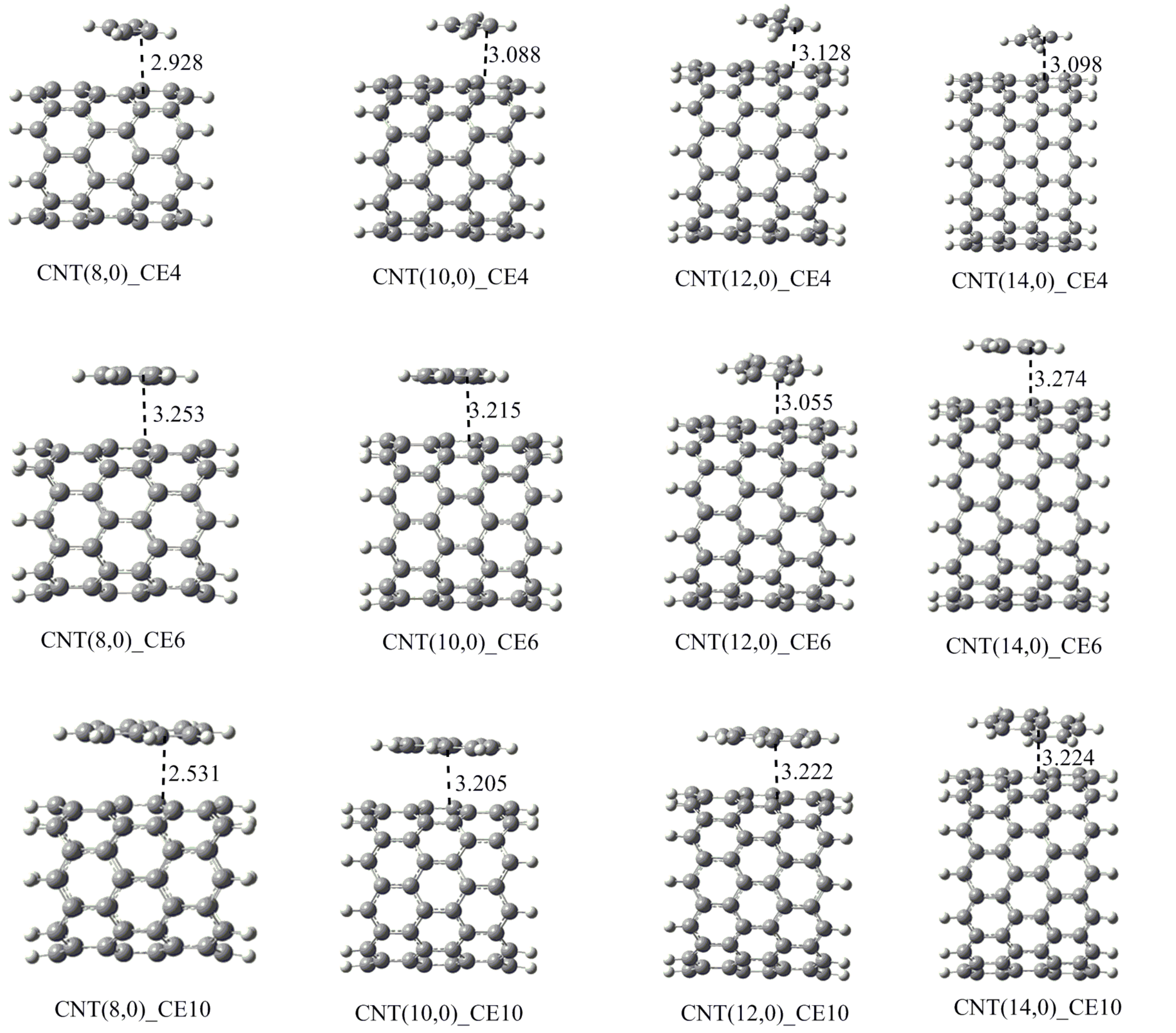


**Figure S13:** Optimized geometries of the complexes of zigzag CNTs with CE*n* at ONIOM(M06-2X/6-31G*:AM1) level and the nearest distance (Å) between them.

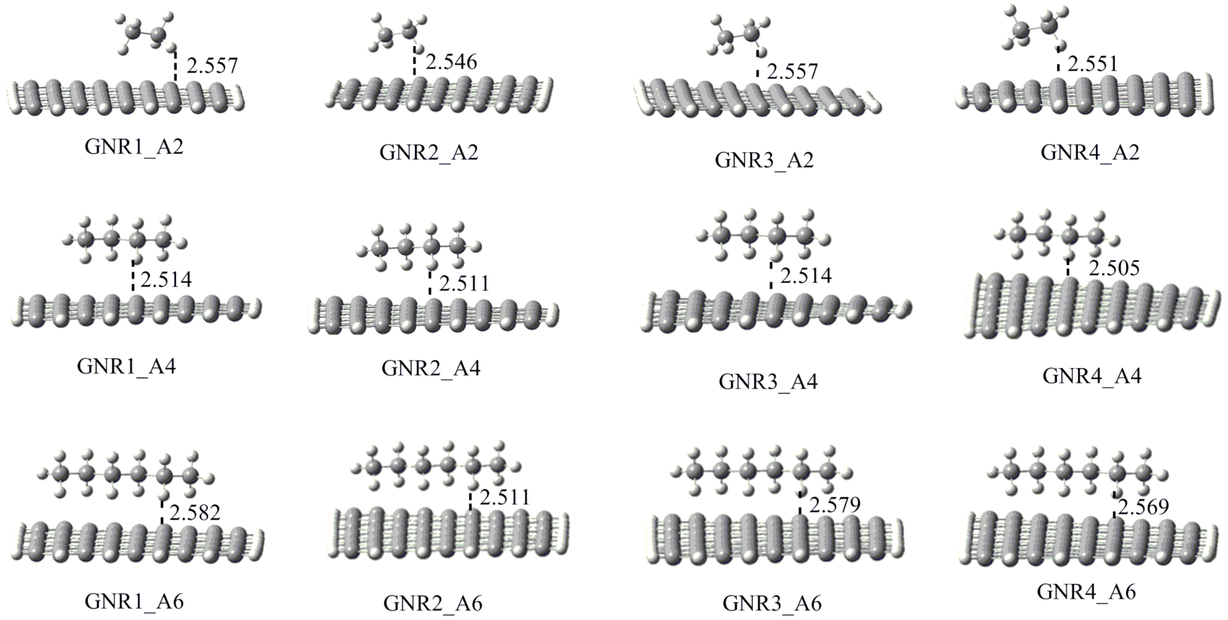


**Figure S14:** Optimized geometries of the complexes of GNRs with A*n* at ONIOM(M06-2X/6-31G*:AM1) level and the nearest distance (Å) between them.

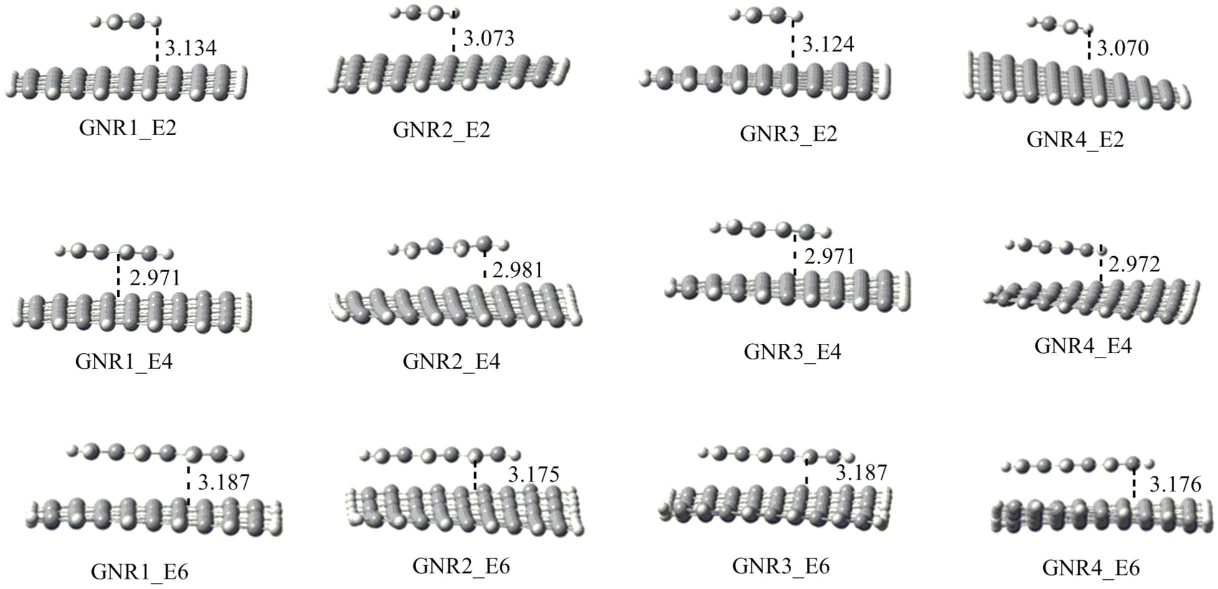


**Figure S15:** Optimized geometries of the complexes of GNRs with E*n* at ONIOM(M06-2X/6-31G*:AM1) level and the nearest distance (Å) between them.

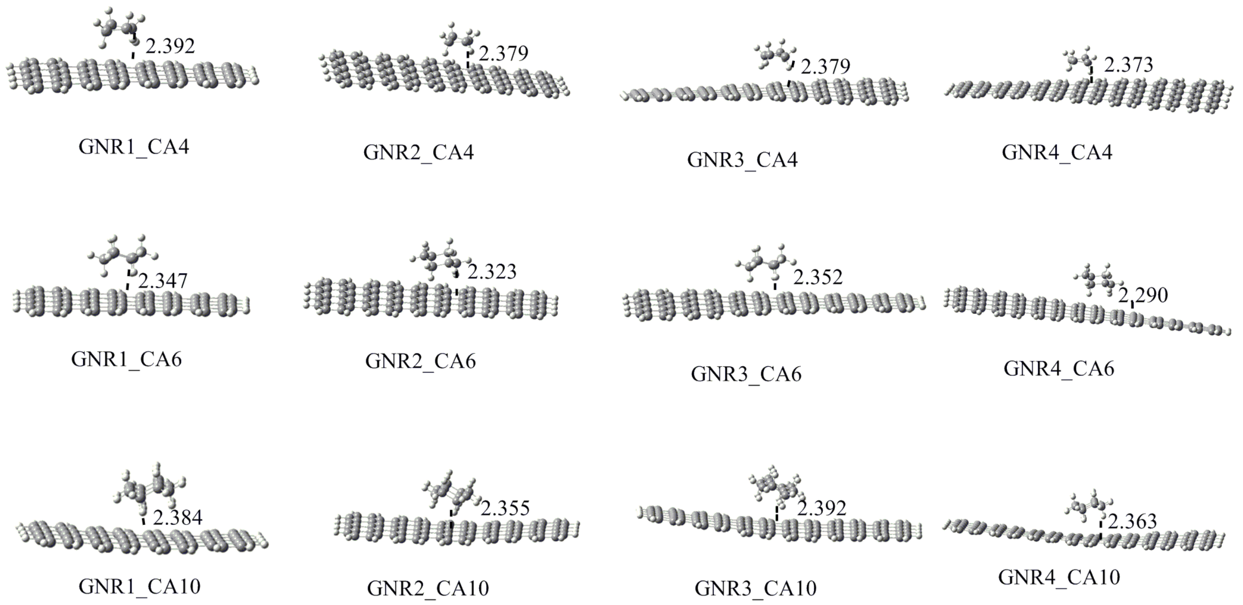


**Figure S16:** Optimized geometries of the complexes of GNRs with CA*n* at ONIOM(M06-2X/6-31G*:AM1) level and the nearest distance (Å) between them.

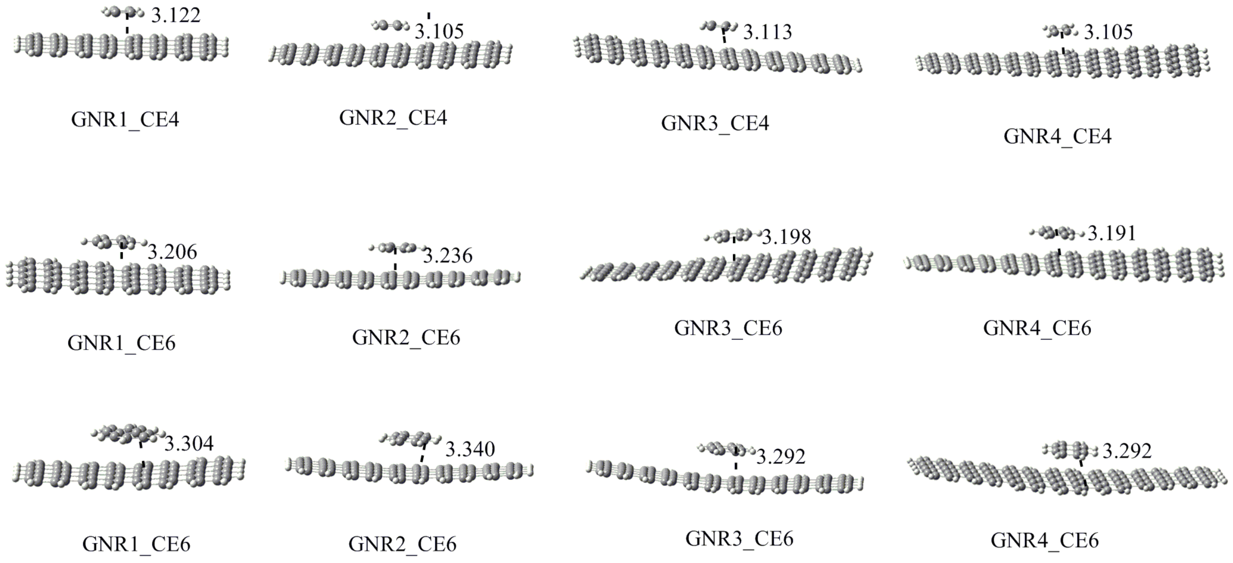


**Figure S16:** Optimized geometries of the complexes of GNRs with CE*n* at ONIOM(M06-2X/6-31G*:AM1) level and the nearest distance (Å) between them.

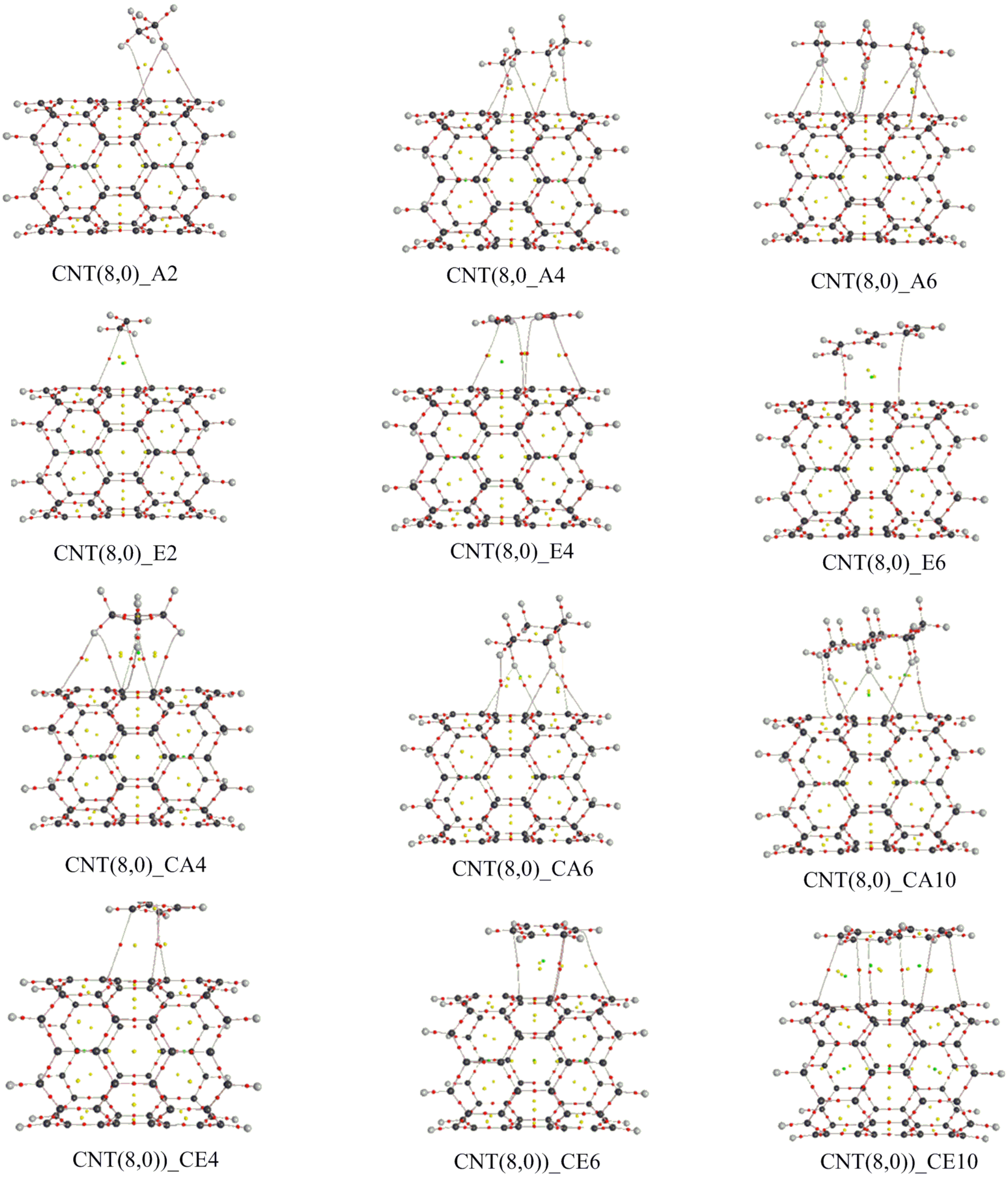


**Figure S17:** Atomic positions and critical points of CNT complexes with hydrocarbons obtained at M06-2X/6-31G* level. BCPs are represented by red color dots.

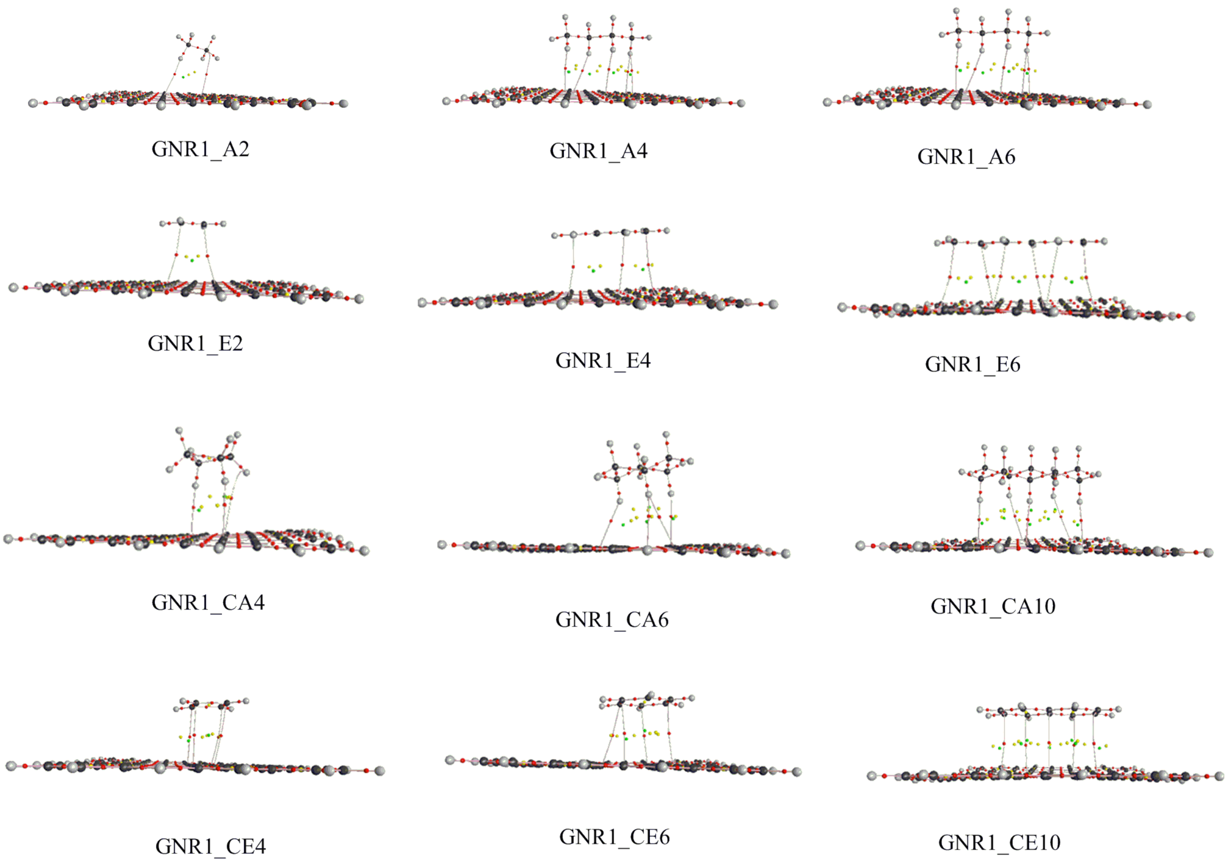


**Figure S18:** Atomic positions and critical points of CNT complexes with hydrocarbons obtained at M06-2X/6-31G* level. BCPs are represented by red color dots.
